# Comparative Study of Machine Learning and System Identification for Process Systems Engineering Dynamics

**DOI:** 10.1021/acs.iecr.4c03264

**Published:** 2025-02-12

**Authors:** Akhil Ahmed, Ehecatl Antonio del Rio-Chanona, Mehmet Mercangöz

**Affiliations:** Centre for Process Systems Engineering, Department of Chemical Engineering, Imperial College London, London SW7 2BX, U.K.

## Abstract

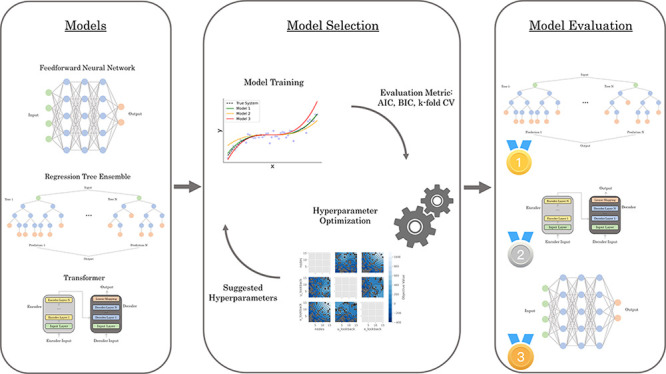

This study provides a comprehensive benchmarking of traditional system identification and modern machine learning (ML) models for the data-driven modeling of dynamical systems, with a focus on process systems engineering (PSE) applications. To achieve this, we deploy AutoSID, an automated end-to-end framework inspired by Machine Learning Operations (MLOps) principles. While AutoSID facilitates model selection, training, validation, and evaluation, its purpose here is to serve as a platform to investigate how well MLOps-inspired tools can be adapted for system identification tasks in PSE. Our investigation includes a comparison of 12 diverse model architectures from the system identification, machine learning, and deep learning literature, evaluated across 11 PSE case studies under varying data regimes. We employ four model search or hyperparameter optimization algorithms and three model selection criteria to ensure a thorough assessment. Our findings highlight the importance of model selection as the crucial step in system identification. Specifically, our results demonstrate the effectiveness of Bayesian optimization with tree-structured parzen estimators (TPE) for balanced model selection, while k-fold cross-validation proves to be a robust metric for performance evaluation during the selection process. In large-scale data scenarios, where performance differences between *k*-fold cross-validation and information criteria are small, information criteria emerge as a computationally efficient alternative. Once the “best” model structure is decided, in terms of model performance, we find that ML models with balanced complexity, such as tree ensemble models, consistently achieve superior predictive accuracy and computational efficiency, outperforming both simplistic and overly complex models. These findings provide actionable insights into model selection and performance evaluation for PSE practitioners and demonstrate the potential of incorporating MLOps-inspired workflows into the system identification process.

## 1 Introduction

Machine Learning (ML) has been extensively applied across diverse fields, including computer vision,^1^ medicine,^2^ and notably natural language processing.^3,4^ While ML has been applied to the modeling of dynamical systems, particularly in engineering contexts, its performance relative to established system identification models has not been systematically evaluated.^5−7^ Data-driven dynamical systems modeling, also referred to as system identification, is a critical task in engineering, enabling the development of model-based control and optimization strategies such as Model Predictive Control. These models are particularly important when first-principles models are unavailable or insufficient to capture system complexity.^8−10^

ML models have shown significant promise for system identification tasks by offering the potential to model complex, nonlinear dynamics.^11−14^ However, the field of system identification has a long history of robust methods developed by the control, statistics, and time-series analysis communities, such as ARX, ARMAX, and state-space models.^15−17^ The integration of ML into this domain raises questions about its advantages over traditional methods and the specific scenarios in which ML-based approaches are preferable. Existing studies exploring these comparisons have yielded mixed results, with some highlighting ML’s potential,^7,18,19^ while others demonstrate traditional methods’ advantages in terms of simplicity and interpretability.^20−22^

Despite these studies, a consistent and rigorous benchmarking methodology remains lacking. Many existing comparisons focus exclusively on deep learning (DL) models,^6,7^ often evaluating them in isolation or alongside a limited set of ML models. While some studies include multiple modeling approaches, the methodologies employed often lack consistency, particularly in terms of model selection strategies, evaluation criteria, and applications considered. In particular, model selection—a critical step in determining the appropriate model structure/hyperparameters—has been inconsistently applied. ML models are frequently evaluated using ad hoc configurations, whereas traditional models are subjected to extensive tuning.^20,21^ An illustrative case is found in a previous study,^20^ where the evaluation of DL models relied on fixed, preselected configurations, while traditional models underwent rigorous model selection. This inconsistency undermines the fairness of comparisons and limits the insights that can be drawn about the relative performance of these approaches.

A promising avenue to address these challenges is the adoption of Machine Learning Operations (MLOps) tools. These tools are designed to automate and streamline the processes of model training, validation, and deployment, enabling efficient workflows for selecting and benchmarking models.^23−25^ While MLOps tools have been extensively applied by ML practitioners, their efficacy for system identification tasks remains underexplored. Instead, these steps are often carried out in an ad-hoc manner. This approach demands substantial engineering effort, incurs major expenses, and creates an inefficiency in the scaling of model-based solutions for the control and optimization of large-scale systems.^26^ This study aims to address this gap by assessing how MLOps-inspired tools can be adapted for system identification. We do this through the use of Automated System Identification (AutoSID), a fully automated end-to-end framework for data-driven modeling of dynamical systems. This framework aims to bridge the gap and streamline processes, akin to those seen in MLOps, for the system identification domain. Specifically, in this paper, our focus is on evaluating these tools’ ability to support benchmarking studies that compare ML and traditional models in PSE applications.

**Figure 1:**
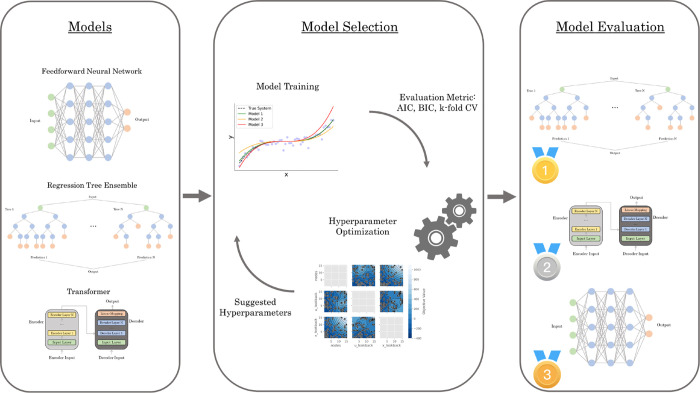
Conceptual depiction of the main steps performed by the AutoSID framework used in this study.

For the above reasons, we propose a thorough comparative benchmarking on the application of traditional system identification and modern ML models for the data-driven modeling of dynamical systems. We selected several promising models from both sets of approaches and evaluated their performance across a spectrum of dynamical systems prevalent in the PSE community. To overcome the limitations outlined earlier, we propose the implementation of a MLOps-inspired end-to-end pipeline for model selection, training, validation, and subsequent testing and comparison, as illustrated conceptually in Figure 1. Specifically, we consider an assortment of different model selection approaches as well as different model selection criteria. Additionally, we utilize a variety of model performance criteria beyond the standard metric of model “accuracy” alone. We also tested the performance of the different models in the small and big data limits.

Our contributions are as follows:

1. A rigorous benchmarking study comparing 12 model architectures, spanning traditional system identification, ML, and DL methods, applied to 11 diverse dynamical systems in PSE.
2. An evaluation of MLOps-inspired workflows tailored for system identification, including their effectiveness in automating model selection and performance evaluation.
3. A systematic analysis of model performance across small- and large-data regimes, considering both accuracy and computational efficiency.
4. Insights into the strengths and limitations of traditional and ML-based models in practical engineering contexts, with actionable recommendations for practitioners.

## 2 Background and Methodology

In this section, we detail the entire end-to-end framework, AutoSID, employed in this study, while also providing the relevant background information and literature. In Section 2.1, we start by giving a high-level overview of AutoSID including the steps performed, results generated, and important implementation details. In subsequent sections, we investigate each of the steps in detail. Specifically, in Section 2.2, we start by exploring the PSE case studies which are used to simulate and generate data for the investigation. This is followed by Section 2.3, where we detail all of the data-driven models that are used to perform system identification. We then consider the two primary steps employed in the framework: model selection, which is detailed in Section 2.4, and finally, model performance evaluation, which is presented in Section 2.5.

### 2.1 AutoSID: End-to-End Framework

AutoSID is the MLOps-inspired workflow that we use in this study to systematically evaluate and benchmark combinations of systems, data-driven models, and model selection strategies. Specifically, the results generated by AutoSID allowed us to investigate the following:

1. Compare four different model selection search (hyperparameter optimization) algorithms.
2. Compare three different model selection criteria.
3. Compare the performance of 12 different models from the system identification, ML and DL literature when fitting these models to 11 different data sets obtained from nonlinear dynamical systems prevalent in the PSE literature.
4. Compare the above when using a different number of training samples in order to assess the performance of the models in the small to big data regimes.

**Figure 2:**
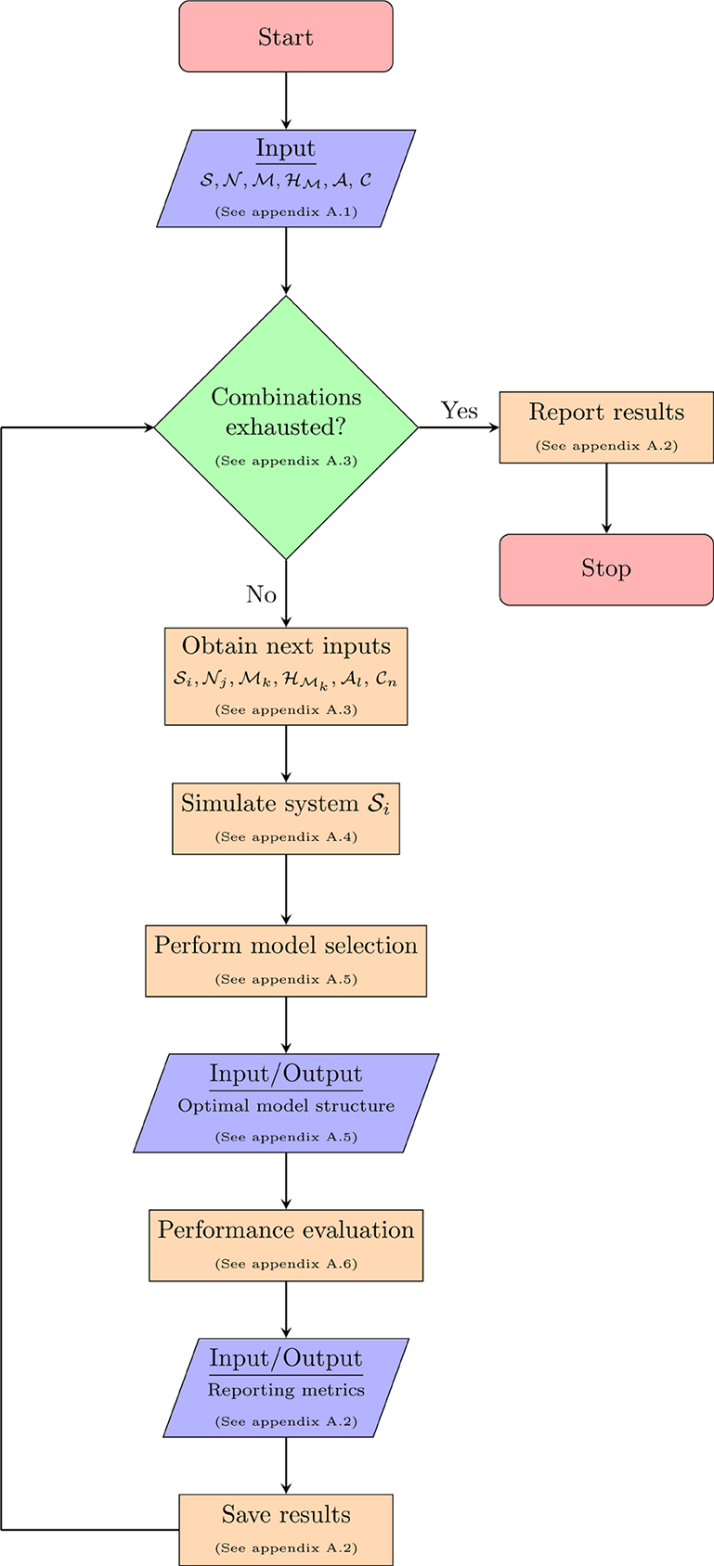
AutoSID: Proposed end-to-end framework was used in this study.

A high-level flowchart of the proposed end-to-end framework is shown in Figure 2. A detailed breakdown of the steps is presented in Appendix A, which includes relevant implementation details. Please note that Figure 2 contains references to the relevant sections in Appendix A for each of the steps. The key steps in the framework are described below:

1. **Input Initialization**: The framework begins by defining six input sets: dynamical systems , sample sizes , data-driven models , hyperparameters , hyperparameter optimization algorithms , and selection criteria . These inputs specify the configurations used for simulation, training, model selection, and evaluation. See Appendix A.1 for more details.
2. **Iterative Evaluation**: Each step of the framework is repeated for all combinations of inputs, ensuring an exhaustive exploration. Each iteration involves selecting a specific instance from one of the input sets, with the results stored for subsequent analysis. See Appendix A.3 for more details.
3. **Simulation of Dynamical Systems**: For the selected system , time-series data is generated, with a portion allocated for training and the rest reserved for testing. The number of training samples  is determined in this step. See Appendix A.4 for more details.
4. **Model Selection**: The framework performs model selection by optimizing the hyperparameters  of the chosen data-driven model  using a specified search algorithm  and selection criterion . The optimal configuration and the time taken for selection are recorded. See Appendix A.5 for more details.
5. **Performance Evaluation**: The model with the optimal configuration is trained on the full training data set, and its performance is evaluated on both training and testing data sets. Metrics such as training/testing error and computational time are computed and stored. See Appendix A.6 for more details.
6. **Results Storage**: For each iteration, the results—spanning the selected system, data-driven model, optimal configuration, training/testing errors, and computational times—are saved in a database for comprehensive analysis. See Appendix A.2 for more details.

The iterative approach ensures that all input combinations are explored, facilitating a robust comparison of models, hyperparameter optimization methods, and performance metrics under varying conditions. This process is parallelized for computational efficiency, leveraging high-performance computing resources.^27,28^

### 2.2 PSE Dynamical Systems

In this section, we provide an overview of the 11 PSE dynamical systems used in this study to generate data for the system identification task. Table 1 provides a summary of these systems, including the rationale for their inclusion and highlighting the specific system identification challenges they present. Detailed information about each system, including the governing equations, parameter values, and an in-depth discussion of the complexity of their dynamics, along with relevant literature, can be found in Appendix B.

**Table 1:** Summary of All of the Dynamic Systems Used in This Study to Generate the Time-series Data for Identification

system	number of states	number of inputs	reasons for inclusion
second-order system	2	1	describes many physical processes; oscillatory responses; dampening variations
continuous stirred tank reactor (CSTR)	2	1	highly nonlinear; potentially unstable; displays bifurcations, output multiplicity, chaotic behavior, and self-sustained oscillations
CSTR in series with recycle	4	4	magnifies nonlinearities of CSTR; interacting mass and thermal capacities; long-time dependencies and time delays
four tank system	4	2	standard benchmark for identification; strongly interacting tank liquid capacities; displays minimum phase and nonminimum phase behavior
distillation column	9	2	quintessential distributed parameter system in PSE; accurate dynamic modeling remains challenging; multiple steady states and possible instabilities
multistage extraction cascade	10	2	distributed parameter system; complex dynamics due to multiphase equilibrium and mass transfer; wide range of dynamic behaviors depending on configuration
extraction cascade with reaction	20	2	reaction magnifies complexities of extraction cascade; challenges include slow reaction kinetics, sustained oscillations, and time delays; dynamics further complicated by competing phenomena
fluidized biofilm sand bed reactor	16	5	complex bioprocess with reaction dynamics, mass transfer, and large recycle loops; competing dynamic effects; may exhibit limit cycles and sustained oscillations
shell and tube heat exchanger	24	4	fundamental chemical engineering unit operation; may exhibit pressure build-ups, phase changes, time delays and system instability
free-radical polymerization	3	4	nonlinear reaction dynamics; multiple steady states (“gel” effect); industrial relevance in polymer manufacturing; dynamics studied extensively in the literature
plantwide process system	12	5	large-scale process with multiple components and interactions; recycle loop introduces time delays and dependencies; “snowball” effect demonstrates chaotic dynamics and plantwide identification and control challenges

#### 2.2.1 Data Generation

To thoroughly evaluate the performance of data-driven models under varying data availability scenarios, we designed a data generation procedure that produces both interpolation and extrapolation conditions. Each system under consideration is modeled by its corresponding set of nonlinear ordinary differential equations, discretized and simulated using CasADi and the CVODES solver in Python.^29^ Rather than relying on a single operating condition, we simulate each system over a range of input trajectories to excite its various dynamic modes.

Specifically, we generate a total of 40,000 samples per system by applying 20 random step inputs. These steps vary between predefined lower and upper bounds unique to each system, ensuring a broad exploration of the system’s state space. Details on these bounds and the input, **u** ∈ ^*n*_*u*_^, and output, **y** ∈ ^*n*_*y*_^, variables for each system are provided in Appendix B. We introduce additive white Gaussian noise into the outputs (**y**) to achieve a signal-to-noise ratio of 60 dB, reflecting realistic measurement conditions.

**Figure 3:**
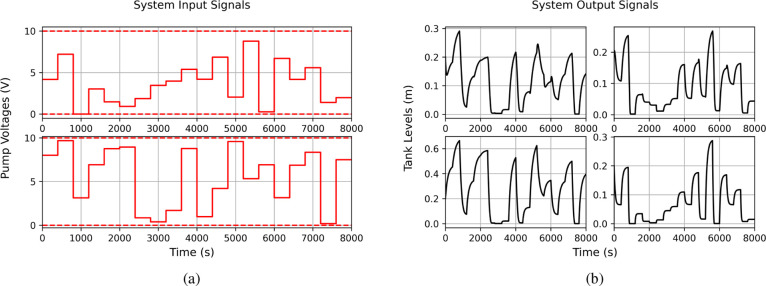
(a): Input signals used to excite the four tank system. (b): System response (output signals) of the tank levels. Noise removed for ease of visualization.

Figure 3 illustrates a representative example of the input and output signals for the four-tank system (see Appendix B.4 for more details). As this figure demonstrates, the data generation procedure ensures diverse operating conditions by simulating the system across a range of inputs.

From this comprehensive data set, we select different training set sizes, to analyze model performance across a spectrum of data richness. For instance, using only 500 samples for training results in a limited and potentially nonrepresentative data set. In this scenario, the test set largely falls outside the training data range, imposing a demanding extrapolation challenge. Conversely, when we train on 20,000 samples, the training set covers a wide range of operating conditions, resulting in a mixture of interpolation (for conditions seen in training) and some extrapolation.

**Figure 4:**
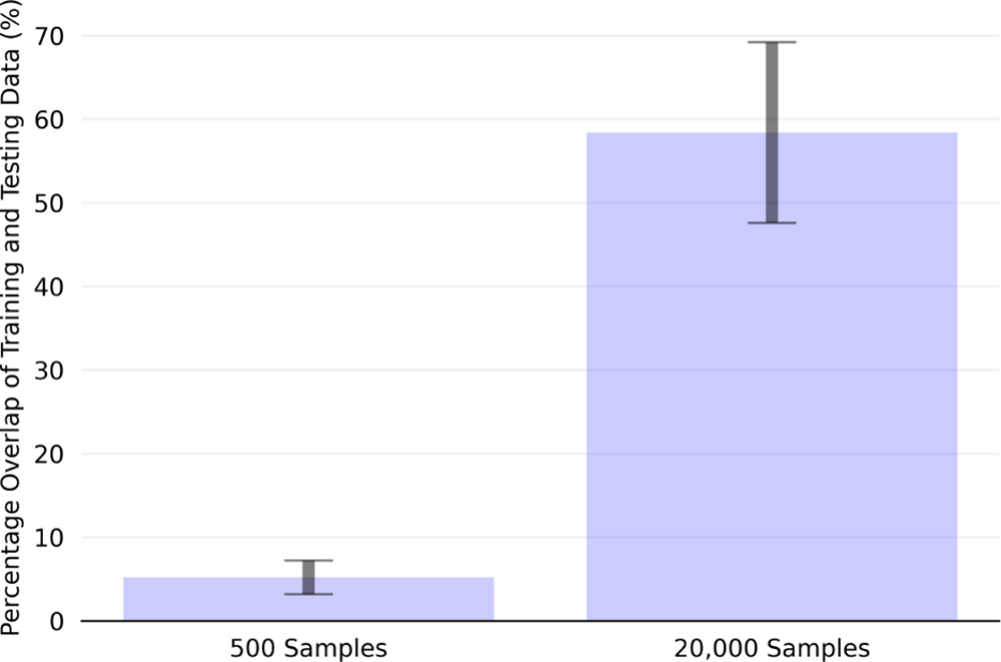
Average and standard deviation of the percentage overlap of the training and testing data sets across all of the systems.

To quantify the extent of interpolation versus extrapolation in these scenarios, we introduce Figure 4, which shows the average percentage overlap between training and testing distributions for the 500-sample and 20,000-sample cases (the focus of our results in Section 3.3). On average, there is only about a 5% overlap with 500 training samples, confirming a strong extrapolation task. With 20,000 training samples, this overlap increases to approximately 60%, indicating a mix of interpolation and extrapolation. This design closely mirrors conditions commonly encountered in industrial system identification campaigns, where data availability and richness vary widely.

By systematically varying the number of training samples, we can assess how different models perform under both extremely challenging (predominantly extrapolative) conditions and more data-rich scenarios. These experiments ensure that our framework evaluates models under realistic and diverse conditions. The results of these tests, focusing on the 500-sample and 20,000-sample scenarios, are presented and analyzed in Section 3.3.

### 2.3 Data-Driven Models

In this section, we detail all 12 data-driven models that are employed in this study to fit the generated input-output data. These range from classic system identification models to more nascent developments from the fields of ML and especially DL. Particular focus is given to the possible hyperparameters for each data-driven model class so that readers are aware of the number of hyperparameters and their choices for each of them (these can be found in Appendix C). This becomes important in the crucial step of model selection as will be discussed in Section 2.4. A brief discussion of the data-driven models and relevant sources of literature is also presented.

All data-driven models were implemented using a mixture of Python and MATLAB libraries/toolboxes. This included PyTorch and scikit-learn for the ML and DL models, and the System Identification Toolbox in MATLAB for the system identification models. Please note that this is separate to the PSE dynamical system simulations from Section 2.2 which are discretized and simulated using CasADi and the CVODES solver.

#### 2.3.1 Autoregressive with the Exogenous Input (ARX) Model

The Autoregressive with Exogenous Input (ARX) model belongs to a class of polynomial time-series models commonly used in system identification. ARX models represent dynamic systems in discrete time by expressing system output as a linear combination of past outputs and inputs, enabling efficient parameter estimation using linear least-squares methods.^17,8^ This simplicity has historically made ARX models highly practical, particularly in resource-constrained environments, as popularized by researchers like Glad and Ljung.^8,15^

Despite their linearity, ARX models remain effective for systems operating within ranges where linear assumptions hold true and often outperform more complex ML and DL models in constrained scenarios. This advantage is attributed to their resilience against overfitting and simplicity in parametrization.^20,21,6^ Notably, these models contrast with modern ML/DL approaches that rely on computationally intensive parameter estimation methods.

The ARX model’s hyperparameters consist of two primary components: the autoregressive window lengths for the output and input data (model orders). This minimal hyperparameter set simplifies model selection, as methods such as grid search are sufficient. Table 13 summarizes the hyperparameter ranges explored in this study.

#### 2.3.2 Autoregressive Moving Average with the Exogenous Input (ARMAX) Model

The Autoregressive Moving Average with Exogenous Input (ARMAX) model extends the ARX model by incorporating a moving average component to account for the system noise. This additional flexibility makes ARMAX models particularly suited for identifying dynamical systems subject to significant noise, a common occurrence in industrial applications.^17,30,31^

Like ARX models, ARMAX models rely on linear combinations of past outputs and inputs for system representation but with the added ability to model noise as a moving average of white noise. This study employs the pseudolinear regression approach available in MATLAB’s System Identification Toolbox, which solves the parameter estimation problem efficiently using multistage linear least squares.^15^

The hyperparameters of ARMAX models include the autoregressive window lengths for both output and input data, as in ARX models, along with the window length for the moving average component. This simple hyperparameter structure facilitates exhaustive model selection, such as a grid search. The explored hyperparameter ranges are detailed in Table 14.

#### 2.3.3 State-Space Model

State-space models, widely adopted by the early system identification community, represent dynamical systems using first-order difference equations rather than the wide autoregressive windows of polynomial time-series models.^15,8,32^ This approach offers distinct advantages, particularly for control applications, as it aligns naturally with the field of “Identification For Control” and the analysis of multivariable systems.^33,34,16^ Additionally, state-space representations allow for the integration of physical insights into models, forming the foundation for many “gray-box” modeling approaches.^8,15^

State-space and polynomial time-series models are mathematically equivalent in many respects, and polynomial models (e.g., ARX, ARMAX) are often converted into state-space forms for control-specific analysis.^15^ Parameter estimation for state-space models benefits from efficient subspace methods, avoiding iterative error minimization commonly required by ML/DL models.^15,32,35^ However, these methods can yield less predictive models, necessitating further refinement through iterative techniques. This additional refinement step makes state-space parameter estimation more time-consuming compared with polynomial models.

The primary hyperparameter for state-space models is the number of states (model order). This simplicity, combined with efficient parameter estimation, allows for exhaustive model selection approaches, such as grid search. Table 15 outlines the range of model orders considered in this study.

#### 2.3.4 Tree-Partitioned Linear Model

Tree-partitioned linear models, also known as Local Linear Model Trees (LOLIMOT),^36−39^ are a straightforward extension of ARX models, categorized under Nonlinear ARX (NLARX) models. NLARX models extend ARX models by incorporating nonlinear mappings of past inputs and outputs, enabling a better representation of nonlinear dynamical systems.

Tree-partitioned linear models approximate nonlinear systems through piecewise linear models. They achieve this by partitioning the input space into a binary tree, where each node contains a locally linear ARX model estimated using efficient linear regression methods (see Section 2.3.1). The number of tree nodes, a hyperparameter, determines model fidelity with more nodes capturing finer nonlinear details. During the prediction, the tree structure facilitates efficient identification of the relevant linear model for a given input.

Widely adopted in the identification community,^36,5^ tree-partitioned linear models balance accuracy and efficiency, making them suitable for modeling a variety of nonlinear systems.^40,38,39^ Their efficient parameter estimation methods result in relatively short training times, supporting their use in real-time applications.^40,37^ Additionally, their simple structure supports robust hyperparameter optimization.^36^ However, their locally linear nature can complicate gradient-based control strategies,^38,39^ and they share ARX models’ limitations when encountering unseen input-space regions during testing.^36^

The hyperparameters for tree-partitioned linear models include the autoregressive window lengths for outputs and inputs (as in the ARX models) and the number of tree nodes. Table 16 summarizes the hyperparameter ranges used in this study.

#### 2.3.5 Single-Layered Sigmoid Network Model

Single-layered sigmoid networks, a subset of NLARX models, are notable for their specialized applications in system identification and control tasks. Although they share similarities with Feed Forward Neural Networks (FFNNs) that may also employ sigmoid activation functions, they are often treated as a distinct category due to their widespread adoption and tailored algorithms within the system identification community.^41,36^ These networks introduce nonlinearity into ARX models using a single sigmoid network layer, balancing improved modeling capability with shorter training times—a critical advantage for real-time applications.^9,42,43^

Single-layered sigmoid networks are particularly effective in capturing nonlinear dynamics that challenge standard ARX models.^44,45^ While their parameter estimation relies on iterative error minimization algorithms similar to those used in ML/DL models, their simplicity (fewer parameters) enables faster convergence and the use of second-order optimization techniques. In this study, MATLAB’s fmincon function is utilized for parameter estimation, a method that is often impractical for overparameterized ML/DL models.

The hyperparameters for single-layered sigmoid networks include the autoregressive window lengths for outputs and inputs (as in ARX models) and the number of sigmoid nodes in the single layer. These parameters, along with their ranges, are detailed in Table 17.

#### 2.3.6 Single-Layered Wavelet Network Model

Single-layered wavelet networks, a class of NLARX models, share similarities with single-layered sigmoid networks but are often preferred for their unique properties and specialized parameter estimation techniques. The work of^41^ introduced a noniterative, efficient parameter estimation algorithm tailored for wavelet networks.

Wavelet functions offer the distinctive ability to localize in both frequency and time, making them particularly effective for modeling complex nonlinearities.^41,46^ This capability has contributed to their broad application across diverse domains, including large-scale process systems and solar photovoltaic cells.^46,47^

The hyperparameters for single-layer wavelet networks include the autoregressive window lengths for outputs and inputs (as in ARX models) and the number of wavelet nodes in the single layer. These parameters and their ranges are summarized in Table 18.

#### 2.3.7 Regression Tree Ensemble Model

Decision tree ensemble models are widely used in ML for regression and classification tasks, demonstrating exceptional performance in time-series forecasting across diverse domains.^48,22,49^ Their effectiveness often surpasses that of basic statistical models and even deep neural networks, aligning with early findings by Bates and Granger, who highlighted the superior forecast accuracy of ensemble methods over individual models.^50,51^

Despite their computational demands—requiring the training of multiple models and the efficient combination of their outputs—regression tree ensembles maintain feasibility through the inherent efficiency of decision tree algorithms.^52,53,48,54^ This makes them suitable for applications such as real-time control, which are often impractical for other ensemble methods.^54^ However, their adoption within the system identification community remains limited, possibly due to the discontinuous nature of decision tree models, a challenge shared with tree-partitioned linear models (see Section 2.3.4). Such discontinuities can hinder model-based control strategies reliant on gradient information.

A critical hyperparameter for tree ensembles is the fitting method, which determines how individual trees are built and combined. The two most common methods, bagging and boosting, have shown conflicting performance results in time-series forecasting,^22^ making this a key consideration in our study. Overall, the hyperparameter set for regression tree ensembles includes five main choices, as detailed in Table 19.

#### 2.3.8 Feed Forward Neural Network (FFNN) Model

FFNNs are the first DL models considered in this study. Comprising an input layer, one or more hidden layers, and an output layer, FFNNs compute outputs by applying a nonlinear activation function to a weighted sum of inputs at each neuron. For time-series forecasting and system identification tasks, FFNNs process historical input-output data in an autoregressive manner, effectively making them a complex class of NLARX models, as noted by Ljung.^6,5^

FFNNs are highly effective across various applications, including time-series forecasting and system identification.^6,55,56^ However, their multilayered architectures pose challenges, particularly in the form of prolonged training times and susceptibility to overfitting.^6^ Parameter estimation typically involves iterative prediction error minimization algorithms, such as the ADAM optimizer used in this study and implemented via PyTorch.^57,58^ These training requirements, combined with the models’ extensive parametrization, make FFNNs data-intensive and prone to overfitting when insufficient training data is available. Consequently, careful model selection and hyperparameter optimization are essential to avoid unnecessarily large and complex architectures.^59^

The hyperparameter set for FFNNs includes six main choices, detailed along with their value ranges in Table 20.

#### 2.3.9 Vanilla Recurrent Neural Network (RNN) Model

Vanilla RNNs are DL models specifically designed to handle sequential data by processing inputs in a time-dependent manner.^60,59^ Unlike FFNNs, which propagate information strictly forward, RNNs maintain a hidden state that captures temporal dependence within the data. This feature makes RNNs analogous to Nonlinear State-Space models, as described by Ljung,^6^ due to their ability to represent internal system states rather than relying on autoregressive input-output windows.

RNNs excel in applications involving variable-length sequences, such as time-series forecasting and natural language processing.^60,59^ Like FFNNs, they are highly parametrized and trained using iterative prediction error minimization algorithms, specifically backpropagation through time.^61^ This study employs the ADAM optimizer for training the RNN models.

However, vanilla RNNs face significant challenges in addition to those common to DL models, such as overfitting, long training times, and data-intensive requirements. A critical limitation is the vanishing gradient problem, where gradients diminish as they propagate backward through time, hindering the training of deep RNNs.^62^ To overcome this, advanced variants like long short-term memory (LSTM) networks and gated recurrent units (GRUs) have been developed to handle long-term dependencies more effectively.^63^ These will be discussed in Sections 2.3.10 and 2.3.11.

The hyperparameter set for vanilla RNNs includes six main choices, detailed along with their value ranges in Table 21.

#### 2.3.10 Long Short-Term Memory RNN Model

LSTM networks, a specialized type of RNN, were designed to address the limitations of vanilla RNNs in capturing long-term dependencies within sequential data.^62^ Unlike vanilla RNNs, LSTMs incorporate gating mechanisms that enable the selective updating, retention, and forgetting of information from previous time steps, effectively mitigating the vanishing gradient problem.^62,59^ This capability makes LSTMs particularly suited for identifying slowly evolving nonlinear dynamics in system identification tasks.^6^

Similar to vanilla RNNs, LSTMs are trained using the ADAM optimizer with back-propagation through time. However, they share common DL challenges, including susceptibility to overfitting, lengthy training times, and data-intensive requirements. In scenarios involving exceptionally lengthy dependencies, such as those caused by substantial time delays, LSTMs may struggle to effectively model these features effectively. In such cases, emerging models like transformers (discussed in Section 2.3.12) may provide a more suitable alternative.^64^

The hyperparameter set for LSTM models includes seven main choices, detailed along with their value ranges in Table 22.

#### 2.3.11 Gated Recurrent Unit (GRU) RNN Model

GRU models are a type of RNN designed to address the vanishing gradient problem inherent in vanilla RNNs. GRUs simplify the gating mechanisms used in LSTM networks by combining the forget and input gates into a single “update gate”, which controls the retention of the previous hidden state and the addition of new candidate activations. Additionally, GRUs include a “reset gate” to manage how much of the previous hidden state is considered when computing the candidate activation.^63^

With fewer parameters than LSTMs, GRUs are generally faster to train, making them a practical choice for resource-constrained applications or real-time tasks such as control.^60^ However, their simplified architecture may limit their ability to capture long-term dependencies compared to LSTMs, potentially making them less effective in scenarios involving slowly evolving dynamics.^60,65^

The hyperparameter set for GRUs is identical to that of LSTMs, as detailed in Table 22.

#### 2.3.12 Time-Series Transformer Model

Transformers have revolutionized natural language processing by excelling at capturing long-term contextual dependencies through their self-attention mechanism.^3,4^ Their success in tasks like language translation and text summarization, as seen in Large Language Models such as ChatGPT, has also highlighted their potential for analyzing other sequential data types, including time series.^3,64,66^ This makes transformers a compelling inclusion in this study.

The self-attention mechanism is the cornerstone of transformers’ proficiency in handling sequential data. Unlike traditional sequence models such as RNNs, which process sequences step-by-step, transformers allow each position in the input sequence to attend dynamically to all other positions. This enables the model to capture complex, long-range dependencies within the data, making transformers particularly effective for time-series tasks.

Despite their strengths, transformers have notable limitations. Their performance often hinges on access to large-scale data sets, which can be a challenge in fields like system identification where data is frequently scarce. Additionally, their computational demands are significant. The self-attention mechanism, while powerful, scales quadratically with sequence length, making the training of transformers computationally intensive, particularly for longer sequences.^3^

For this study, we adopt the time-series transformer architecture proposed by,^64,66^ designed specifically for forecasting tasks. Model parameters are estimated using the ADAM optimizer.

The hyperparameter set for transformers includes seven main choices, detailed along with their value ranges in Table 23.

### 2.4 Model Selection

In this section, we consider the most important step of the proposed framework: model selection. This involves the selection of a specific model structure for the models described in Section 2.3, taking into account their possible hyperparameters and the available training data. It represents the crux of the challenge of selecting an appropriate model for the problem at hand but is oft-ignored as discussed in Section 1.^67−69^ The primary goal is to compare well-constructed and optimized models to (a) minimize bias toward any specific model type and (b) evaluate models at their best possible performance within the given data and training time constraints. It consists primarily of two main steps. First, a formal algorithm must be defined to search over the possible hyperparameters of a model, which we refer to as the model search. This will be discussed in more detail in Section 2.4.1. Second, when searching over the possible model structures, evaluation criteria must be defined for the goodness-of-fit, which accounts for the well-established bias-variance trade-off inherent in training.^48^ These model selection criteria will be discussed in Section 2.4.2.

#### 2.4.1 Model Search

Model search is the process within model selection in which a formal algorithm is used to explore the hyperparameter configurations of a model. While the criteria for evaluating the “best” model structure—the objective function—is critical, this is discussed separately in Section 2.4.2. Here, we focus exclusively on search algorithms.

The growing complexity of hyperparameter spaces, particularly in DL, has made model search a computational challenge. Training a single DL model can take hours, and when this is multiplied across various hyperparameter configurations, the model search often becomes a bottleneck in model selection. This issue has driven advancements in AutoML, leading to various open-source and commercial tools for practitioners.^23−25,70^ Efficient search algorithms are thus essential for practical model selection.^71−73^

This computational challenge contrasts sharply with traditional system identification models such as ARX and ARMAX models. For such models, exhaustive brute-force searches over the possible hyperparameters are not only possible but recommended.^8,36,9^ Of course, the trade-off made is the lack of flexibility of the models in comparison to the ML and DL models. Whether this trade-off is beneficial is the topic of this paper.

In this study, we evaluate and compare four model search (hyperparameter optimization) algorithms selected for their balance of state-of-the-art innovation, pragmatic utility, and benchmark relevance. Each algorithm is discussed in detail in the following sections.

##### 2.4.1.1 Grid Search

Grid search is an exhaustive hyperparameter optimization algorithm widely used for its simplicity and effectiveness in scenarios with simple model structures and fast training times.^74,72,70,75^ The algorithm explores a predefined grid of hyperparameter combinations, training and evaluating the model at each grid point using a chosen evaluation metric (discussed in Section 2.4.2). By systematically testing all combinations, the grid search identifies the configuration that achieves the best performance based on the evaluation metric. Despite its straightforwardness, grid search is computationally intensive, particularly for models with numerous hyperparameters, large value ranges, and extended training times, as is typical for many ML and DL applications.^75,76^ However, its exhaustive nature provides a complete view of the hyperparameter space, which can be valuable for understanding how performance varies across different settings.^74,72^ In this study, a grid search serves as a benchmark method, offering a reference point for comparing the efficiency and effectiveness of more advanced hyperparameter optimization algorithms.

##### 2.4.1.2 Random Search

Random search is a hyperparameter optimization algorithm that selects hyperparameter combinations randomly from a predefined search space, rather than exhaustively evaluating all combinations as in grid search.^73,76^ This approach can be more computationally efficient, particularly for large and complex hyperparameter spaces, as it requires fewer evaluations to explore diverse configurations. Empirical studies have shown that random search often outperforms grid search under fixed computational budgets, making it especially effective for DL models.^73,76^ By sampling hyperparameters randomly, a random search increases the likelihood of identifying better-performing configurations with fewer iterations. However, as with any randomized algorithm, there is a risk of overlooking critical regions of the hyperparameter space.^77,73^ This highlights the need to balance exploration and exploitation carefully in hyperparameter optimization.

##### 2.4.1.3 Evolutionary Search

Evolutionary search algorithms have gained attention in hyperparameter optimization, particularly for addressing expensive optimization tasks. However, their reported success has been mixed, as highlighted in recent literature.^78,72,79,80^ Among these algorithms, the Covariance Matrix Adaptation Evolution Strategy (CMAES) has emerged as one of the most rigorous and effective approaches for hyperparameter optimization.^81,82,78^ CMAES is well-suited for navigating complex, highly dimensional search spaces. Unlike other evolutionary algorithms such as Genetic Algorithms^83,84^ or Particle Swarm Optimization,^85,86^ CMAES employs an adaptive covariance matrix to adjust the step size of candidate solutions dynamically. This mechanism balances exploration and exploitation, enabling efficient traversal of the search space while accounting for correlations and nonlinear relationships between hyperparameters.^81,78^ CMAES has been shown to outperform other evolutionary strategies in terms of convergence speed and solution quality.^82^ Despite its theoretical strengths, empirical evaluations of CMAES and other evolutionary algorithms for hyperparameter optimization remain relatively limited.^78^ Furthermore, direct comparisons between evolutionary strategies and other established hyperparameter optimization methods are scarce. This study addresses this gap by employing CMAES to assess its effectiveness and positioning it within a broader context of hyperparameter optimization approaches.

##### 2.4.1.4 Bayesian Optimization

Bayesian optimization is widely regarded as the state-of-the-art for tackling expensive, data-driven optimization problems, particularly in hyperparameter optimization.^87−89^ Following the seminal work of Bergstra et al.,^73^ Bayesian optimization has become a cornerstone of hyperparameter search, with the Tree Parzen Estimator (TPE) emerging as a highly effective approach. In this study, we employ TPE as the chosen Bayesian optimization method. TPE utilizes a probabilistic model to estimate the performance of different hyperparameter configurations, guiding the search toward promising regions of the search space. By modeling conditional probability distributions of “good” and “bad” hyperparameter configurations, TPE efficiently explores and exploits the search space, making it particularly adept at handling both discrete and continuous hyperparameters. Its adaptability allows TPE to capture complex relationships between hyperparameters. Compared with Gaussian-process-based Bayesian optimization methods, TPE offers distinct advantages. It typically requires fewer evaluations to identify high-performing hyperparameter configurations due to its efficient exploration-exploitation trade-off. Moreover, TPE excels in high-dimensional search spaces by leveraging its tree-based structure, enabling it to navigate these spaces effectively.^73,75^ This capability makes TPE particularly suitable for scenarios in which hyperparameter spaces are both large and complex. TPE’s combination of efficient exploration, adaptability, and robustness in high-dimensional settings solidifies its position as a state-of-the-art method for hyperparameter optimization.^71−73,70^

#### 2.4.2 Model Selection Criteria

While the literature on hyperparameter optimization is rich with diverse search methods, the effectiveness of these approaches depends critically on the quality of the model selection criteria used to identify the “best” model structure. These criteria serve as the objective functions, guiding the optimization process.

Model selection criteria are essential for balancing the trade-off between bias and variance. This involves finding a model that is sufficiently complex to capture patterns in the data (low bias) while avoiding overfitting to ensure good generalization to unseen data (low variance).^48^ For example, relying solely on the training data set’s mean squared error (MSE) risks selecting overly complex models prone to overfitting. To address this, various approaches have been developed, including resampling strategies such as k-fold cross-validation for estimating generalization performance,^48^ and probabilistic measures like the Akaike Information Criterion (AIC) and Bayesian Information Criterion (BIC) for balancing complexity and goodness-of-fit.^68,90,91^ These criteria help mitigate overfitting, assess generalization, and facilitate meaningful model comparisons.

In this study, we examine and compare three widely used model selection criteria from the system identification and ML/DL literature. Each is detailed in the following sections.

##### 2.4.2.1 *K*-Fold Cross-Validation

*k*-Fold cross-validation is a resampling strategy designed to provide a more robust model selection criterion than full-sample metrics like the MSE of the training data set.^48,92^ It involves partitioning the data set into *k* equally sized folds, training the model on *k* – 1 folds, and evaluating it on the remaining fold. This process is repeated *k* times with each fold serving as the validation set once. The performance metric, typically the validation set MSE, is averaged across all folds to estimate overall model performance.

This approach reduces the risk of overfitting or underfitting by assessing model performance across multiple subsets of the data and makes efficient use of the available data set, as every observation is used for both training and validation.^48,92^ However, k-fold cross-validation can be computationally expensive, especially for large data sets and/or complex models, as it requires *k* training iterations. The choice of *k* also influences results, with smaller *k* values potentially leading to higher variance and larger *k* values increasing bias and computational costs.

For this study, we employed 5-fold cross-validation, a common variant that balances computational feasibility and robustness. While more thorough analysis using multiple *k* values would have been ideal, computational constraints necessitated this limitation.

Unlike probabilistic measures such as information criteria, k-fold cross-validation offers a direct, data-driven method for model evaluation without assumptions about the underlying data-generating process. This makes it a widely adopted approach in the ML and DL literature.^48,92^

##### 2.4.2.2 Akaike Information Criteria (AIC)

The AIC is a probabilistic model selection criterion that contrasts with resampling strategies like k-fold cross-validation.^68,90^ AIC evaluates models based on their likelihood function while penalizing model complexity:
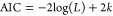
1where *L* is the likelihood function of the model and *k* is the number of model parameters. Models with lower AIC values are considered better in terms of balancing the goodness-of-fit and complexity.

AIC’s primary advantage lies in its ability to navigate the trade-off between model fit and complexity. By penalizing models with more parameters, it discourages overfitting and promotes simpler models. This makes the AIC a useful quantitative tool for guiding model selection.

However, AIC relies on the assumption that the true model is within the set of candidates, which may not always hold. Additionally, like other information criteria, it involves assumptions about the data and the model that may limit its applicability in certain scenarios. For instance, Section 3.2 discusses a situation where AIC’s assumptions may not align with practical outcomes.

Compared to k-fold cross-validation, AIC provides a concise, computationally efficient, single-value summary of model quality. While cross-validation directly assesses model performance on the data set without assumptions, it is computationally expensive, particularly for complex models and/or large data sets.

##### 2.4.2.3 Bayesian Information Criteria (BIC)

The BIC is another example of a probabilistic model selection criteria based on the likelihood function, incorporating a stronger penalty for model complexity than AIC:^68,91^
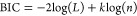
2where *L* is the likelihood function of the model, *k* is the number of model parameters and *n* is the number of samples in the training data set. Like AIC, BIC identifies models with lower values as better but adopts a Bayesian perspective, incorporating prior probabilities on the models.

BIC’s main strength lies in its ability to balance model fit and complexity. Compared with AIC, its stronger penalty for complexity favors simpler models, providing a safeguard against overfitting. Unlike AIC, which assumes that the true model is within the candidate set, BIC selects the model with the highest approximate posterior probability, allowing for a broader exploration of the model space. This Bayesian perspective acknowledges that the true model may lie outside the considered candidates, offering additional flexibility. However, both AIC and BIC share sensitivity to small sample sizes, with BIC being particularly affected due to the log(*n*) term in equation 2.^93^ This makes BIC more sensitive in the small-sample limit.

Finally, BIC shares the same advantages and disadvantages as AIC when compared to k-fold cross-validation: a single-value metric that does not require an expensive resampling strategy but does so at the expense of making several assumptions.

### 2.5 Performance Evaluation and Comparison

The evaluation and comparison of model performance constitute the final step of AutoSID. Following the model selection phase, the optimal model structure is identified and trained on the entire training data set. During this process, the training MSE and the training times are recorded. Subsequently, the model’s performance is evaluated on the unseen test data set, with the test MSE documented. All results—including training MSE, testing MSE, total training time, and model selection time—are archived for further analysis.

The recorded set of results forms the basis for analyzing the four key points outlined in Section 2.1. The ensuing section of this article, Section 3, is dedicated to presenting and discussing the outcomes derived from this analysis.

## 3 Results and Discussion

In this section, we use the results generated by AutoSID to analyze the four main comparison points outlined in Section 2.1. We start by analyzing these results in logical order as outlined by AutoSID shown in Figure 2, starting with the problem of model selection in Sections 3.1 and 3.2. These sections provide answers to points 1 and 2 in Section 2.1. Finally, once the “best” data-driven model structure is chosen, we compare these models to one another assessing their accuracy in terms of the test MSE as well as the training time when applied to the 11 PSE systems of interest. We also compare their performance in various data limits. This is detailed in Section 3.3. These results address points 3 and 4 in Section 2.1.

### 3.1 Model Selection: Model Search

In this section, we present a detailed comparison of the model search algorithms discussed in Section 2.4.1. The primary focus of our work is on evaluating these algorithms based on two key criteria: the performance/quality of the data-driven model selected, as measured by the test MSE, and the efficiency of the model search process, quantified by the model selection time. Essentially, we are dealing with multiobjective criteria where the goal is to balance these two competing objectives: finding the best-performing model while minimizing the search time. This multiobjective criteria is important to consider, because taking grid search as an example, if performed exhaustively, it theoretically guarantees the discovery of the model with the lowest test MSE. However, this exhaustive approach is often impractical due to its extensive model selection time. Therefore, our comparison aims to identify which search or hyperparameter optimization algorithm best balances the trade-off between achieving a low test MSE and maintaining reasonable model selection times.

Readers may note that the search algorithm’s performance in finding the optimal model depends on the specific selection criteria used. However, because all algorithms are evaluated under the same criteria, we argue that any variations in performance due to criteria biases will average out, allowing us to focus on the intrinsic effectiveness of the search algorithms themselves. To provide a fair comparison, we present the results in normalized terms rather than absolute values, as the absolute performance metrics can vary significantly depending on the model type and data set. For instance, the training time for a transformer model differs vastly from that of an ARX model. Finally, to ensure comparability, all model selection algorithms are constrained to a maximum budget of 200 iterations.

**Figure 5:**
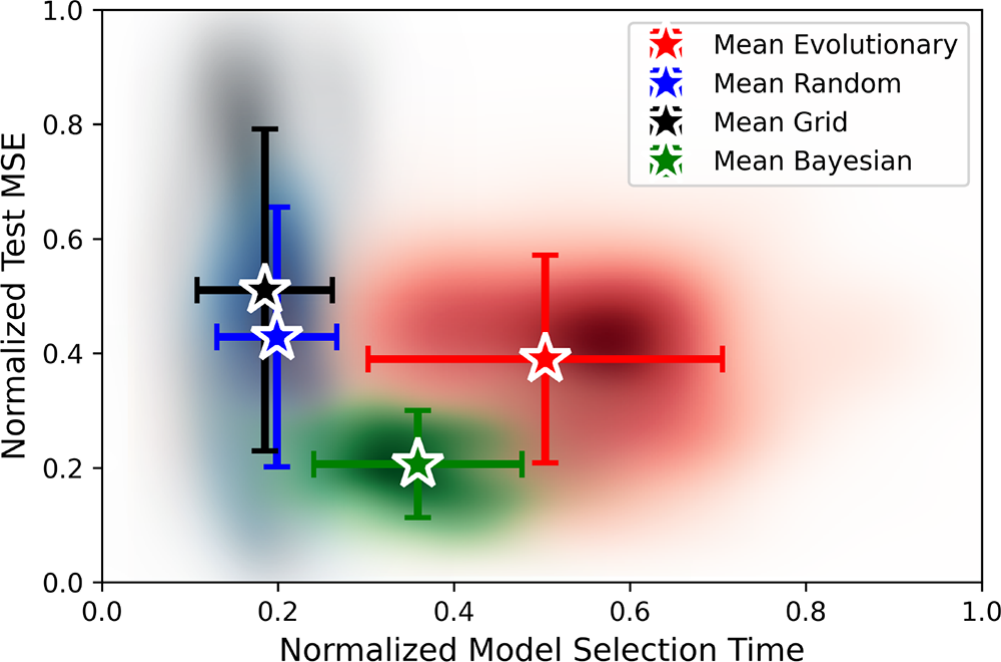
Results depicting the performance of grid search (black), random search (blue), evolutionary search (red), and bayesian optimization (green) in terms of the normalized test MSE of the models selected and the normalized model selection time. Mean results are shown as colored starred markers and the variance in the results is shown with error bars. A colored heatmap underlays all of the plots to show the density of the results around the mean and variance.

Figure 5 illustrates the results of our comparison, with the *x*-axis representing the normalized model selection time and the *y*-axis indicating the normalized test MSE of the models selected. The mean performance for each algorithm is depicted using a colored star-shaped marker, with the color representing the algorithm used. Error bars extending from each star indicate the variance in the performance across different runs. To provide a visual representation of the distribution of results, a heatmap is underlaid on the plot. This heatmap illustrates the density of results for each algorithm, with darker colors signifying regions where results are more concentrated, whereas lighter colors represent areas with sparse data. The findings from this comparison reveal several insights.

First, both grid and random search emerge as the fastest algorithms in terms of model selection time. This efficiency arises from their simplicity; they explore the search space either as a grid or randomly. However, these methods exhibit relatively poor performance in terms of selecting the best model, as evidenced by their higher normalized test MSE compared to other algorithms, with the grid search being slightly worse than the random search. Moreover, the performance of both grid and random search exhibits significant variance in terms of the normalized test MSE as indicated by the large vertical error bars. Specifically, for simpler model structures with smaller search spaces, both algorithms can achieve excellent results. In contrast, for more complex model structures with larger search spaces, these methods often yield suboptimal performance. This variance suggests that while grid and random searches might occasionally identify effective models, their performance is highly dependent on the nature of the model and the complexity of the search space. In general, both methods led to inconsistent results.

Second, we observe that evolutionary search surprisingly performs only marginally better than random search in terms of test MSE, despite requiring significantly more time for model selection. Intuitively, one might expect that the increased computational effort invested in the evolutionary search would lead to a substantial improvement in model performance. However, our empirical results reveal that this expectation is not met in practice. In other words, the trade-off between the time spent and the improvement in test MSE does not favor evolutionary search as one might anticipate.

It is important to note, however, that we evaluated the CMAES algorithm within only the broader category of evolutionary search methods. Other evolutionary algorithms, as indicated by various studies,^83−85^ may potentially offer better performance or a more favorable trade-off between time and model quality. Thus, while our results provide insights into the limitations of CMAES, they do not rule out the possibility that other evolutionary search techniques could be more competitive in different scenarios.

Finally, our results indicate that the Bayesian optimization stands out as the best-performing algorithm. It achieves the lowest test MSE on average, with a narrow variance in performance across different runs. Although Bayesian optimization requires a longer model selection time compared with grid and random search, the superior model quality justifies the additional computational effort. In contrast to the evolutionary search, Bayesian optimization achieves a more favorable balance between model performance and efficiency.

In summary, our results indicate that Bayesian optimization is the most effective model search algorithm overall, offering the best balance between performance and efficiency. For scenarios where computational resources are limited and efficiency is the primary concern, random search emerges as a viable alternative due to its quick execution. Therefore, we recommend Bayesian optimization for most model selection tasks to achieve the best results, while random search can be employed when computational constraints are a significant factor.

### 3.2 Model Selection: Selection Criteria

While the search algorithm employed for model selection is a critical component, the choice of criteria used to evaluate the models is arguably even more pivotal. In this section, we offer a thorough comparison of different model selection criteria, as detailed in Section 2.4.2, through the lens of our experiments and observations. Our focus of this study is on evaluating how these criteria perform across varying sample sizes, leveraging both theoretical insights from the literature and empirical results from our study.

Our investigation is guided by a key insight from the literature: Resampling methods, such as k-fold cross-validation, are known for being assumption-free and generally robust, though they are computationally expensive due to the need for multiple model training. In contrast, information criteria have a strong basis in statistical theory but come with significant assumptions, particularly the requirement for a sufficiently large sample size to yield reliable estimates.

**Figure 6:**
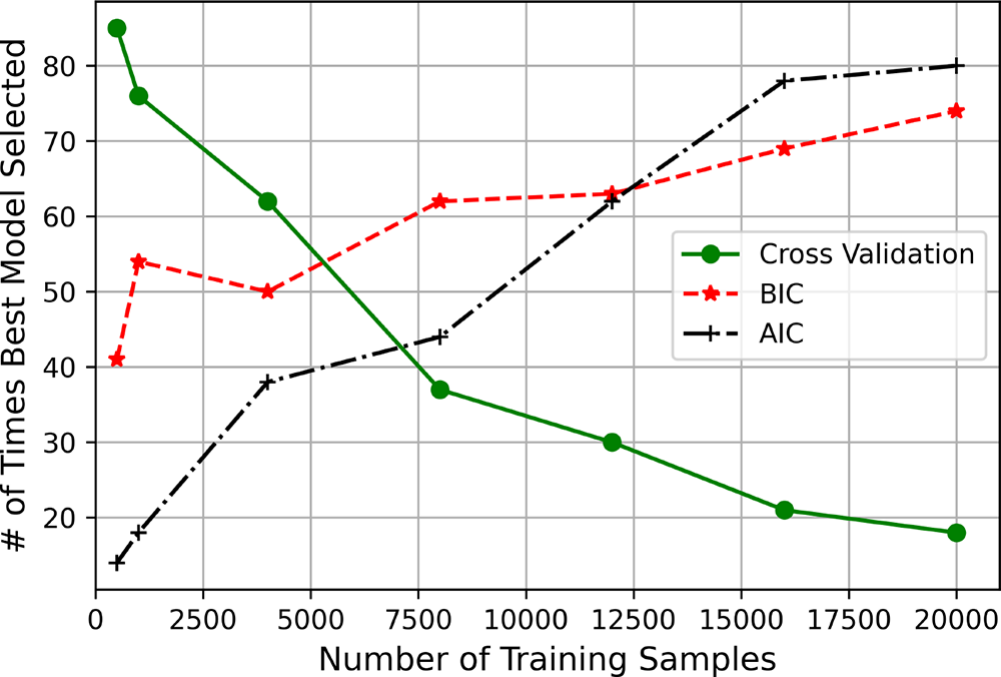
Results depicting the performance of five-fold cross validation (green), AIC (black), and BIC (red) in terms of the “Number of Times The Best Model Was Selected” by each criterion for each model and system combination across various sample sizes.

To assess this concept, we conduct experiments across a range of models and PSE systems, examining the performance of AIC, BIC, and 5-fold cross-validation under varying sample sizes, specifically . For each model and system combination, we use these criteria to select the optimal model structure i.e. one selected by AIC, BIC, and 5-fold cross-validation each. We then fit the selected model structure to the training data. Finally, the performance of these models is assessed on the test data set to give the test MSE. We compare the models chosen by AIC, BIC, and 5-fold cross-validation to determine which criterion selected the “best model” i.e. with the lowest test MSE. The results of our experiments are depicted in Figure 6, which illustrates the performance of AIC, BIC, and five-fold cross-validation across different sample sizes.

When the training data set is small, *k*-fold cross-validation consistently outperforms AIC and BIC in terms of selecting the model with the lowest test MSE. This finding aligns with the theoretical understanding that information criteria are less reliable when sample sizes are insufficient. Information criteria, which are based on asymptotic properties, require large data sets to perform effectively. Conversely, *k*-fold cross-validation’s resampling approach does not rely on such assumptions and thus performs better with limited data in comparison.

However, as the sample size increases, Figure 6 appears to show that information criteria are superior to k-fold cross-validation. This shift is due to the fact that the asymptotic properties assumed by AIC and BIC become more valid in larger data sets, thus enhancing their effectiveness for model selection.

These results are indeed intriguing and provide empirical support for the theoretical findings in the literature. However, they naturally prompt the question: “How much better are these criteria compared to one another?”: The discrete measure of performance depicted in Figure 6 — specifically, the “Number of Times the Best Model Was Selected” — does not fully address this question and can obscure important details.

**Figure 7:**
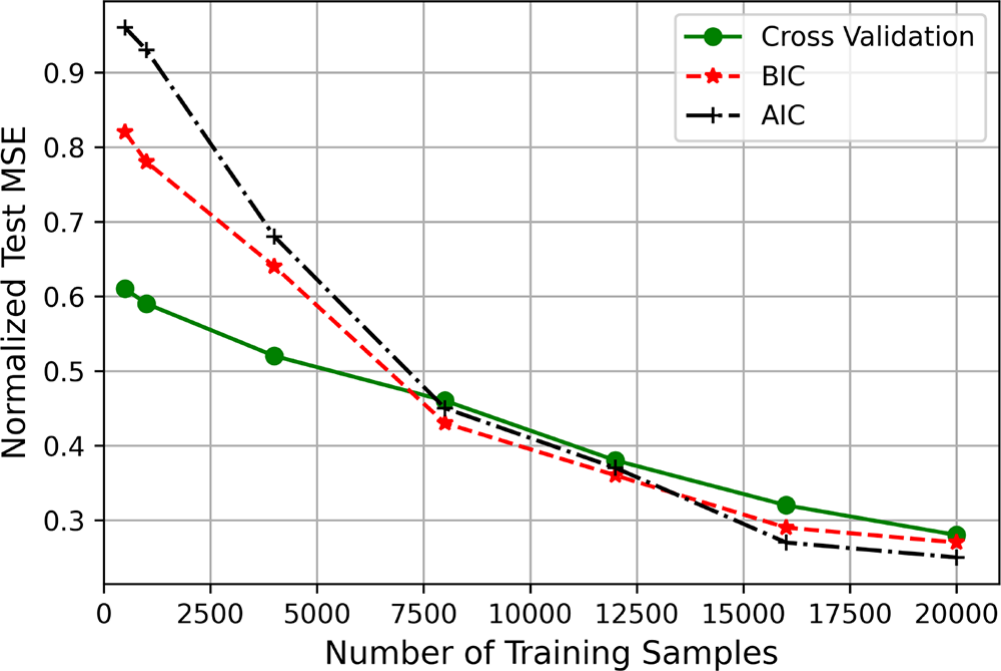
Results depicting the performance of 5-fold cross validation (green), AIC (black), and BIC (red) in terms of the normalized test MSE performance for each model and system combination depending on which selection criteria was used across various sample sizes. This provides a continuous measure of performance, unlike that outlined in Figure 6.

For instance, although Figure 6 might suggest that k-fold cross-validation is a suboptimal choice with large data sets, a deeper examination using the normalized test MSE — a continuous measure of performance — reveals a more nuanced picture. As shown in Figure 7, while AIC and BIC do indeed select models with slightly lower test MSEs compared to k-fold cross-validation in the large-data regime, this difference is marginal at best. In contrast, this difference is much more pronounced in the small-data regime, where k-fold cross-validation significantly outperforms AIC and BIC.

In the context of “big data”, the choice of model selection criterion becomes less critical, as all methods tend to perform similarly well. However, in the small-data regime, the asymptotic assumptions of information criteria appear to hold true empirically, making k-fold cross-validation a more reliable choice. This observation explains why resampling methods like k-fold cross-validation are so prevalent in the ML and DL literature — they are effective across various data sizes, unlike information criteria which are only reliable under specific conditions.

On the other hand, our results also suggest that, for very large data sets in the context of PSE, there may be a practical advantage to using information criteria. Although AIC and BIC offer only marginal improvements over *k*-fold cross-validation, they avoid the computationally expensive process of training the model multiple times, making them a viable option for managing large-scale data.

To summarize, *k*-fold cross-validation is a robust and effective model selection criterion across different sample sizes and is preferred over information criteria in most cases. However, in the big data regime where the performance differences between selection criteria are minimal, information criteria can be a more efficient choice, as they avoid the computational expense of multiple model training associated with *k*-fold cross-validation.

### 3.3 Model Performance Comparison

In this section, we present the critical results of our study, focusing on the performance of the various data-driven models following a rigorous process of model selection as we have shown thus far. Our analysis provides a comprehensive comparison of these models in terms of accuracy and training time, considering both small data regimes (500 training samples) and large data regimes (20,000 training samples). This evaluation highlights the strengths and limitations of each model under different data availability conditions.

#### 3.3.1 Model Comparison in the Small Data Regime

**Figure 8:**
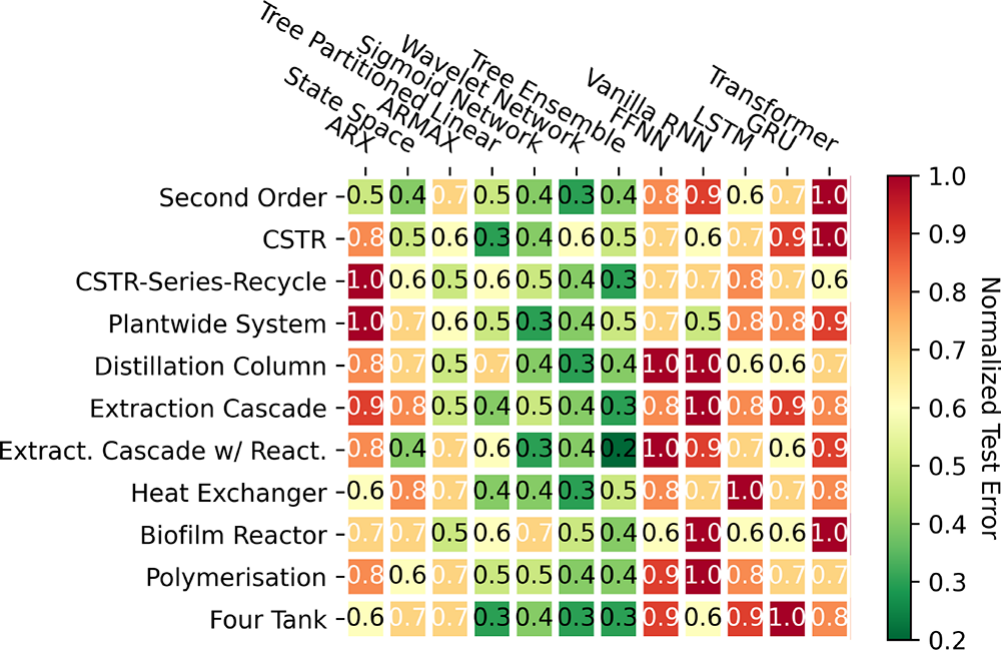
Heat map indicating the normalized test MSE for all of the models tested against all of the systems in the small (500 samples) data regime.

We initiate our comparison by examining the models within the realm of small data, utilizing a mere 500 training samples against a vast testing set of 39500 samples. This scenario presents a formidable prediction challenge, as the limited training data are unlikely to be rich enough to capture the full dynamics of the systems outlined in Section 2.2. This was empirically validated by Figure 4. Illustrated in Figure 8, a heatmap visualizes the normalized test MSE for each data-driven model across various systems (rounded to one decimal place). In this representation, red denotes a high test MSE, indicating poor performance, and green signifies a low test MSE, reflecting good performance. On an absolute scale, all models exhibit poor performance due to the minimal data used for training, leading to pronounced overfitting (as shown later in Figure 11, which shows the results for the four-tank system as an illustrative example). On a relative scale, as depicted in Figure 8, the DL models, ranging from FFNN to transformers, are significantly impacted by this issue, owing to their large parametrization and consequent susceptibility to overfitting. Conversely, simpler models drawn from the system identification literature, such as state space and ARMAX models, demonstrate comparatively better performance. However, excelling above all of these tends to be “moderately” parametrized models, primarily deriving from the ML literature, such as tree ensemble or tree partitioned linear models. This improved performance from the classic system identification and ML models stems primarily from their simpler model structures. This inherently provides a form of “implicit” regularization, circumventing overfitting challenges in the context of limited data which DL models are plagued by.

**Figure 9:**
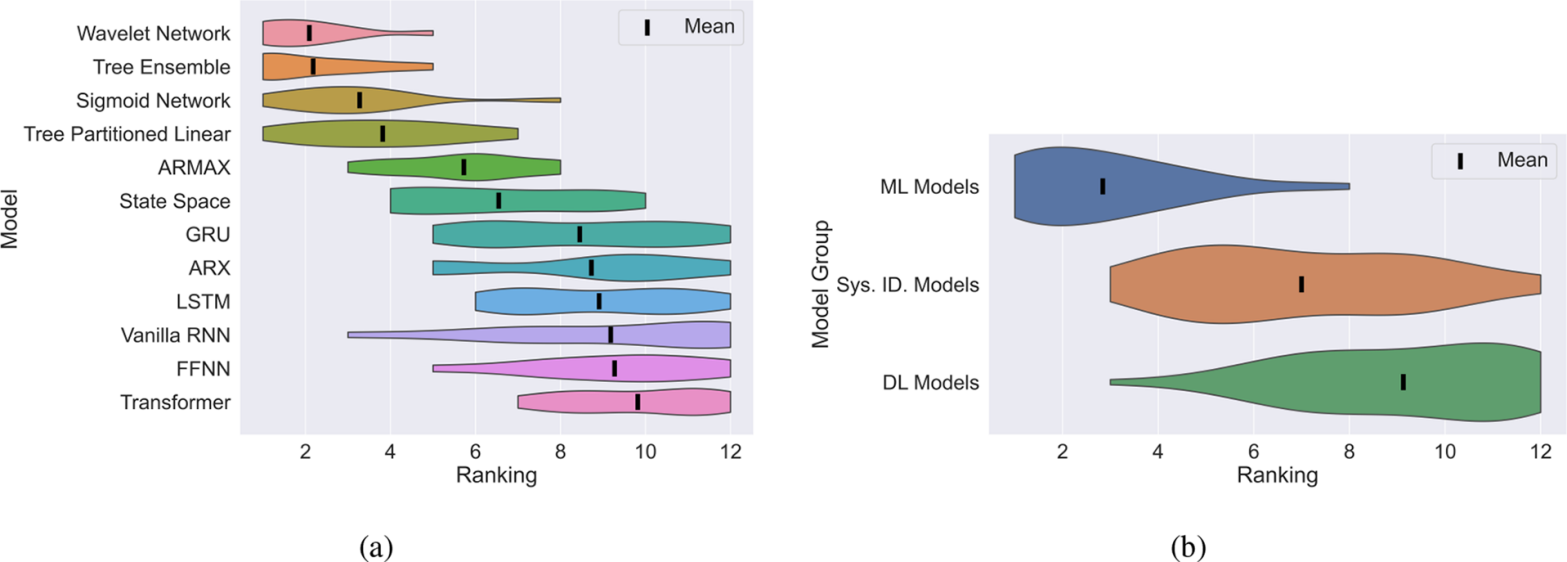
A ridge plot showing the ranking of the models based on their test MSE for the 500 sample case. (a): Individual models ranked. (b): Models were grouped into system identification, ML or DL categories.

To further emphasize this point, we also rank the models from first (best performer in terms of test MSE) to 12th (worst performer in terms of test MSE) given the results in Figure 8. Based on this ranking, Figure 9 depicts a ridge plot showing the mean (black line) and distribution of the ranking of the models (sorted by their mean ranking). Moreover, we group these models into system identification (ARX to ARMAX), ML (Tree Partitioned Linear to Tree Ensemble), and DL (FFNN to Transfomer) models and plot a similar ridge plot for the groupings as shown in Figure 9. Once again, it becomes clear to see that in the small data regime, the “moderately” parametrized ML models perform the best with the top three performing models (wavelet network, tree ensemble, and sigmoid network) all belonging to this class.

**Figure 10:**
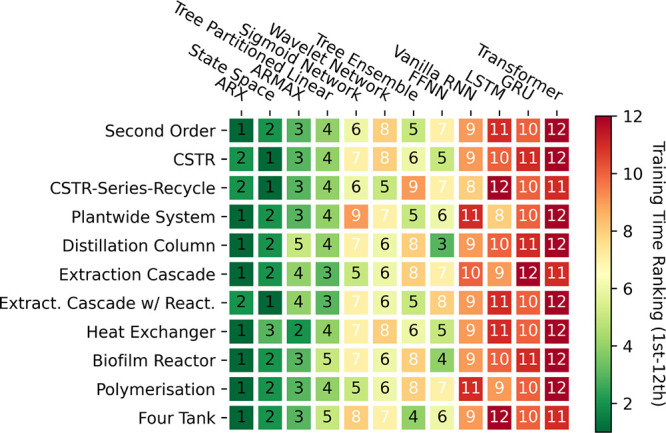
Heat map indicating the training time ranking for all of the models tested against all of the systems in the small (500 samples) data regime.

Transitioning to Figure 10, we observe another heatmap depicting a ranking of training times from first (fastest) to 12th (slowest). Here, the plight is exacerbated for DL models in particular due to their reliance on time-consuming (typically first-order) iterative optimization algorithms, incurring significant computational expense. In contrast, simpler models, especially those linear in structure, such as ARX, ARMAX, and state space models, can be trained with relative simplicity using (pseudo)linear regression methods. On the other hand, moderately parametrized models such as tree ensembles can rely on efficient training algorithms. For instance, recall from Section 2.3.5, single-layered sigmoid networks make use of second-order optimization algorithms which is only possible as they are not overparameterized like their DL counterparts.

**Figure 11:**
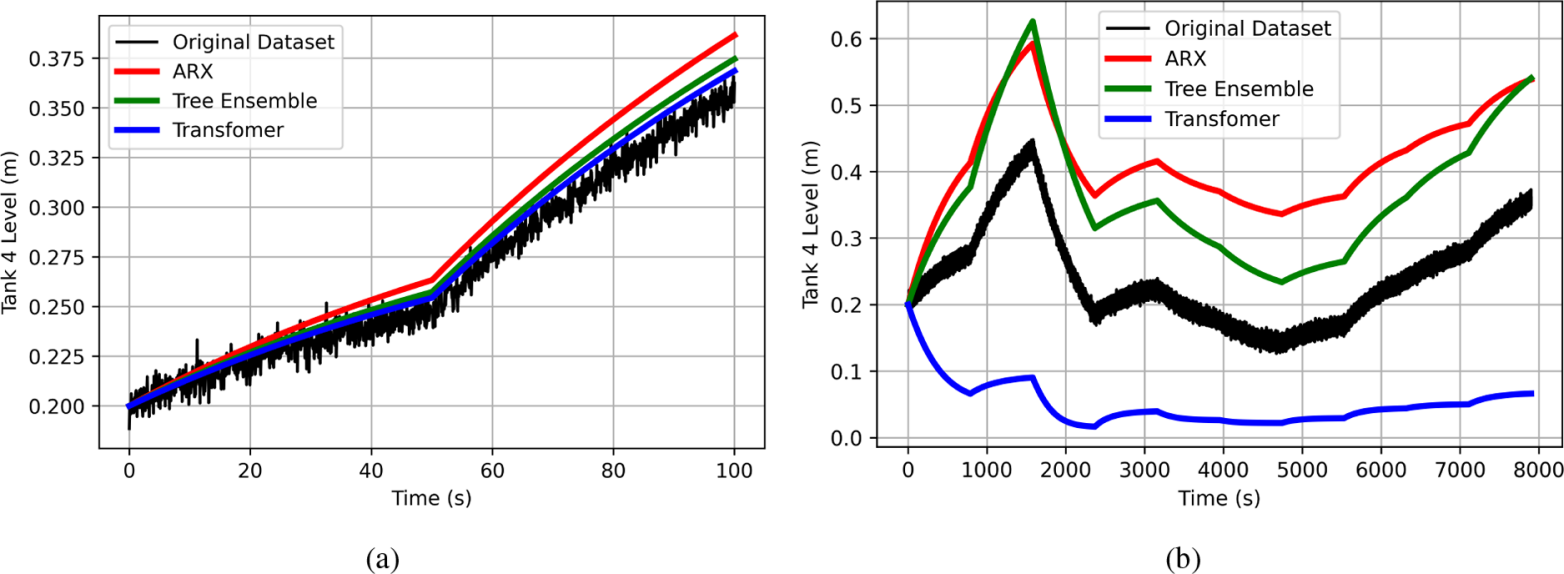
In both figures, the black curve represents the data used for training and testing, and the colored curves indicate the predictions of the respective models (a): Performance of the models during training. (b): Performance of the models during testing.

As an illustrative example, we present the training and testing results for the four-tank system in Figure 11. Specifically, we focus on the prediction of the liquid level in Tank 4 with three distinct data-driven models from the system identification, ML, and DL categories to avoid cluttering the plot. We selected the ARX model from system identification (shown in red), the tree ensemble model from ML (shown in green), and the transformer model from DL (shown in blue). Figure 11 displays the training results, while Figure 11 highlights the testing results. The actual data set is represented in black in both figures. The results clearly underscore the challenges of data-driven modeling with insufficient data. In Figure 11, all models perform well during training, with training MSEs of 2.7 × 10^–3^, 1.1 × 10^–3^, and 6.4 × 10^–4^ for the ARX, tree ensemble, and transformer models respectively. However, as shown in Figure 11, the testing phase reveals significant performance degradation, with all models exhibiting high test MSE values of 0.032, 0.019, and 0.044 for the ARX, tree ensemble, and transformer models respectively. Almost a thousand times higher than the training MSE for the case of the transformer. While the ARX and tree ensemble models fail to reproduce the data accurately, they still show some degree of generalization by capturing similar dynamic behaviors as the true system when subjected to the same input signals. In contrast, the transformer model, which significantly overfitted the training data, not only lacks accuracy but also fails to replicate the expected dynamical behavior of the system, sometimes even exhibiting the opposite effect as the true system when excited by the same input signal.

Combining insights from Figures 8–11, we deduce that in the realm of small data, the adoption of complex, overparametrized models should be approached with caution, as they suffer from overfitting and protracted training times. Notably, the simpler system identification and ML models, such as tree ensembles, emerge as the optimal choices in the small data regime, characterized by good to average predictive performance and fast to moderate training times.

#### 3.3.2 Model Comparison in the Big Data Regime

**Figure 12:**
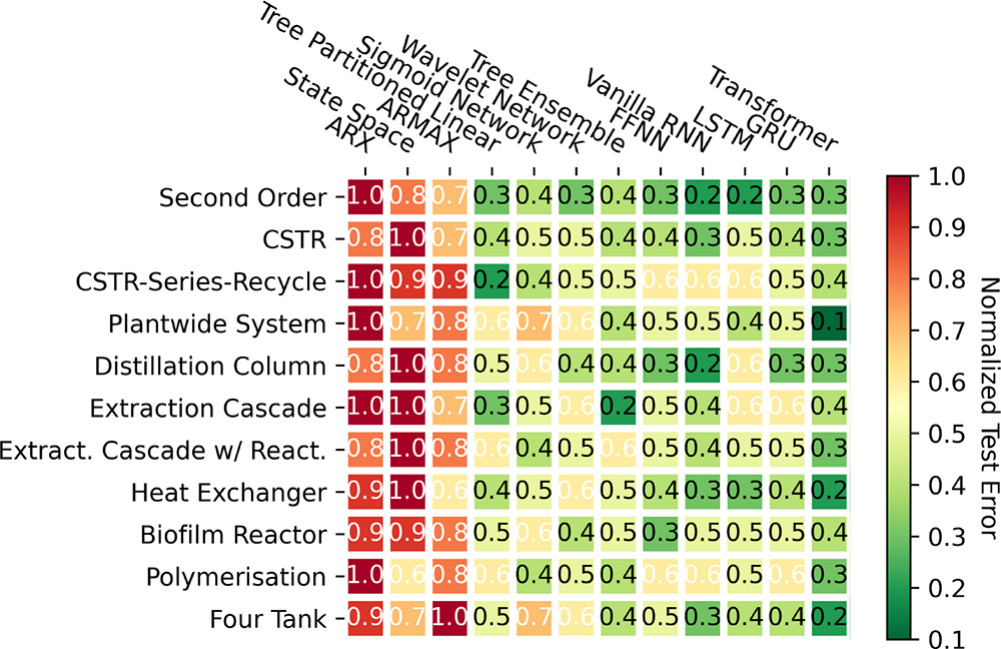
Heat map indicating the normalized test MSE for all of the models tested against all of the systems in the big (20,000 samples) data regime.

In our final analysis, we compare models within the domain of the big data regime, using 20,000 samples for both training and testing. This setting starkly contrasts with that of Section 3.3.1, as now we possess an ample data set likely encompassing the full spectrum of dynamic behaviors exhibited by the considered systems as was empirically validated by Figure 4. As illustrated in Figure 12, which presents a similar heatmap of normalized test MSE, a distinct pattern emerges. ML and DL models, ranging from tree-partitioned linear to transformer models, exhibit superior performance in terms of test MSE. Their nonlinear structures enable them to effectively capture the nonlinear dynamics inherent to the systems under consideration. Conversely, simpler model architectures struggle to perform adequately in this context, barring scenarios involving elementary systems such as the second-order system (although on a relative scale, as depicted in Figure 12, they are still the worst performers). This suggests that, in terms of performance alone, if rigorous model selection is performed, then one should opt for ML and DL models in the big data limit.

**Figure 13:**
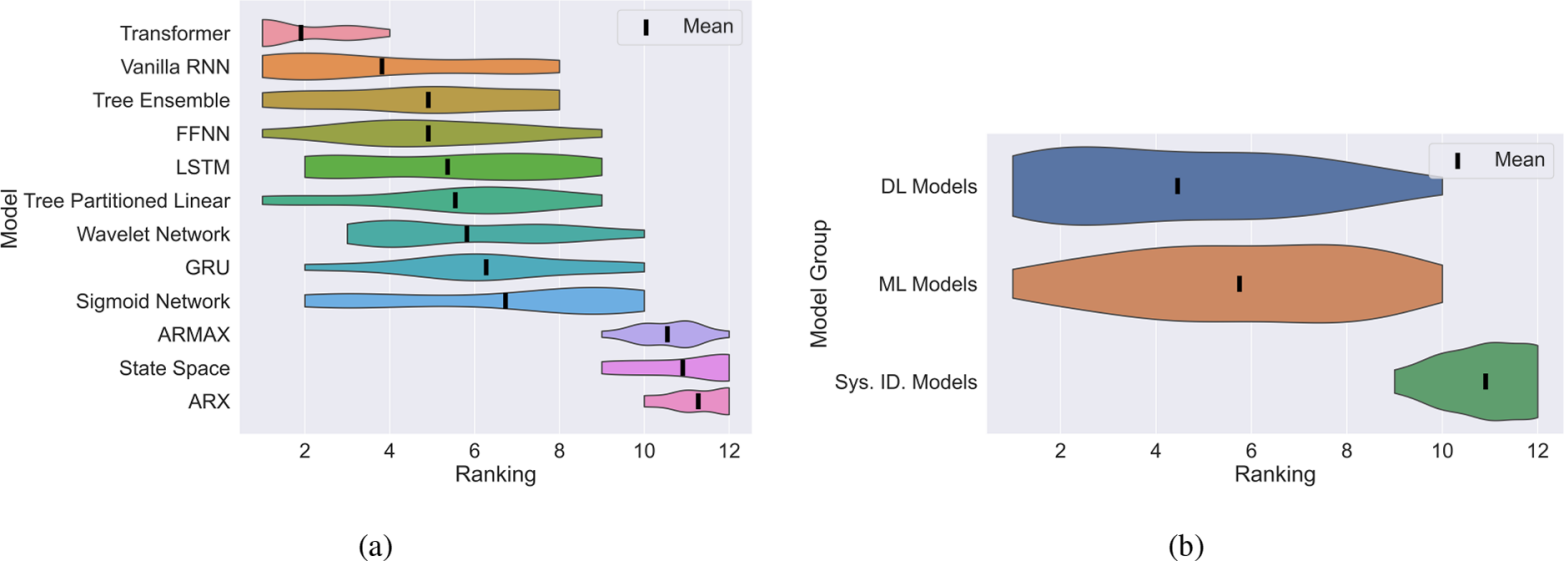
A ridge plot showing the ranking of the models based on their test MSE for the 20,000 sample case. (a): Individual models ranked. (b): Models grouped into system identification, ML or DL categories.

Again, to further emphasize this point, we rank the models as we did previously. This is depicted in Figure 13. Again, Figure 13 ranks these models individually, while Figure 13 groups these models into their different families. In this case, we start to see the dominance of the DL models, with 4 out of 5 of them ranking in the top 5. The ML models also still perform well with their grouped mean performance being similar to the DL models mean performance and with Tree Ensemble models taking the third place in this case.

**Figure 14:**
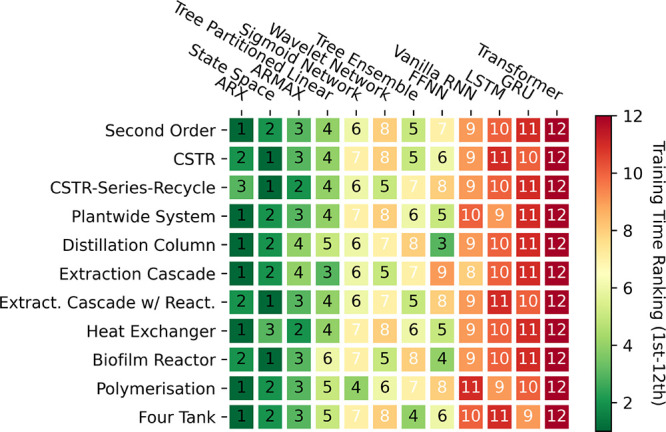
Heat map indicating the training time ranking for all of the models tested against all of the systems in the big (20,000 samples) data regime.

However, despite the apparent prowess of DL models like transformers, Figure 14 unveils a critical drawback associated with their adoption in the big data regime: the substantial training times they entail. In stark contrast, simpler system identification models like ARX models demonstrate rapid training; although as evidenced by Figure 12, they fall short in capturing the nonlinear dynamics of the systems under study.

**Figure 15:**
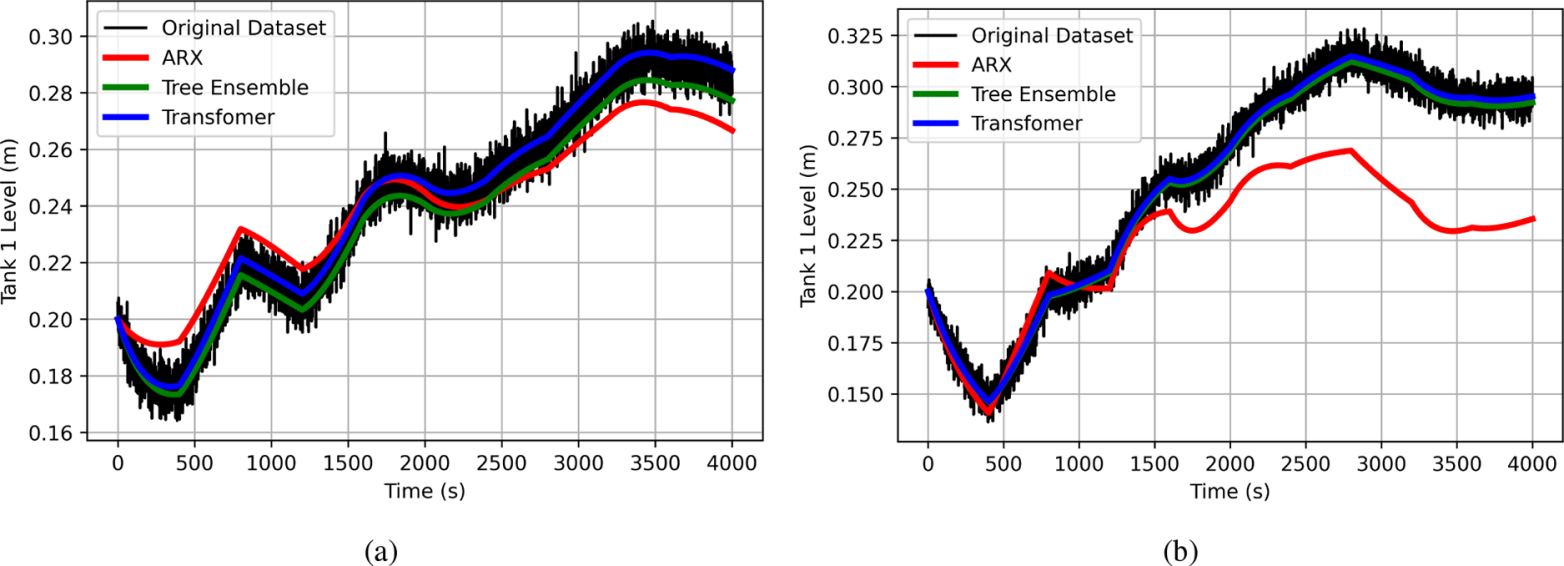
In both figures, the black curve represents the data used for training and testing and the colored curves indicate the predictions of the respective models (a): Performance of the models during training. (b): Performance of the models during testing.

Similar to the previous section, we use the four-tank system as an illustrative example, presenting the training and testing results in Figure 15. This time, we focus on predicting the liquid level in Tank 1 using the same three models as before. Figure 15 displays the training results, and Figure 15 highlights the testing results. In this scenario, the benefits of ML and DL models in the big data context become evident. Both the tree ensemble and transformer models excel during training, achieving MSEs of 1.2 × 10^–4^ and 9.6 × 10^–5^, respectively, and they generalize exceptionally well to the unseen test data set, with corresponding test MSEs of 1.8 × 10^–3^ and 9.2 × 10^–4^. This strong performance is attributed not only to the larger data set but also to the rigorous model selection process, which ensures the selection of robust and parsimonious model structures. On the other hand, the ARX model performs at an adequate to poor level during both training and testing, with MSEs of 7.3 × 10^–3^ and 4.6 × 10^–2^, respectively. However, unlike the tree ensemble and transformer models, the ARX model shows a significantly higher test MSE, indicating a poor generalization. The training results, represented by the red curve in Figure 15, are relatively good but reveal the limitations of ARX models when the system is excited beyond the range for which these linear-in-parameter models are valid. Consequently, they struggle to capture the system’s nonlinear dynamics fully, a challenge that is further exacerbated during testing, leading to the notably higher test MSE. Indeed, ARX models, like other linear-in-parameter system identification models, perform relatively poorly overall in the big data regime (as shown by the heatmap in Figure 12). While they capture the general dynamic behavior of the system in response to input excitation, their accuracy is limited to certain ranges where the linearity assumption holds true.

Again consolidating all sets of results, we realize that a discernible “goldilocks” zone materializes: DL models (ranging from FFNN to transformers in Figures 12 and 14 and as categorized per Figure 13) have excellent predictive capabilities but fall short due to their extensive training times. Conversely, linear-in-parameter system identification models (from ARX to ARMAX in Figures 12 and 14 and as categorized as per Figure 13) exhibit rapid training but lack predictive accuracy. In between these extremes lies a cohort of mostly ML models, ranging from tree-partitioned linear and tree ensemble models in Figures 12 and 14 and as categorized as per Figure 13, which strike the fine balance between training efficiency and predictive accuracy.

#### 3.3.3 Overall Model Performance Rankings

**Figure 16:**
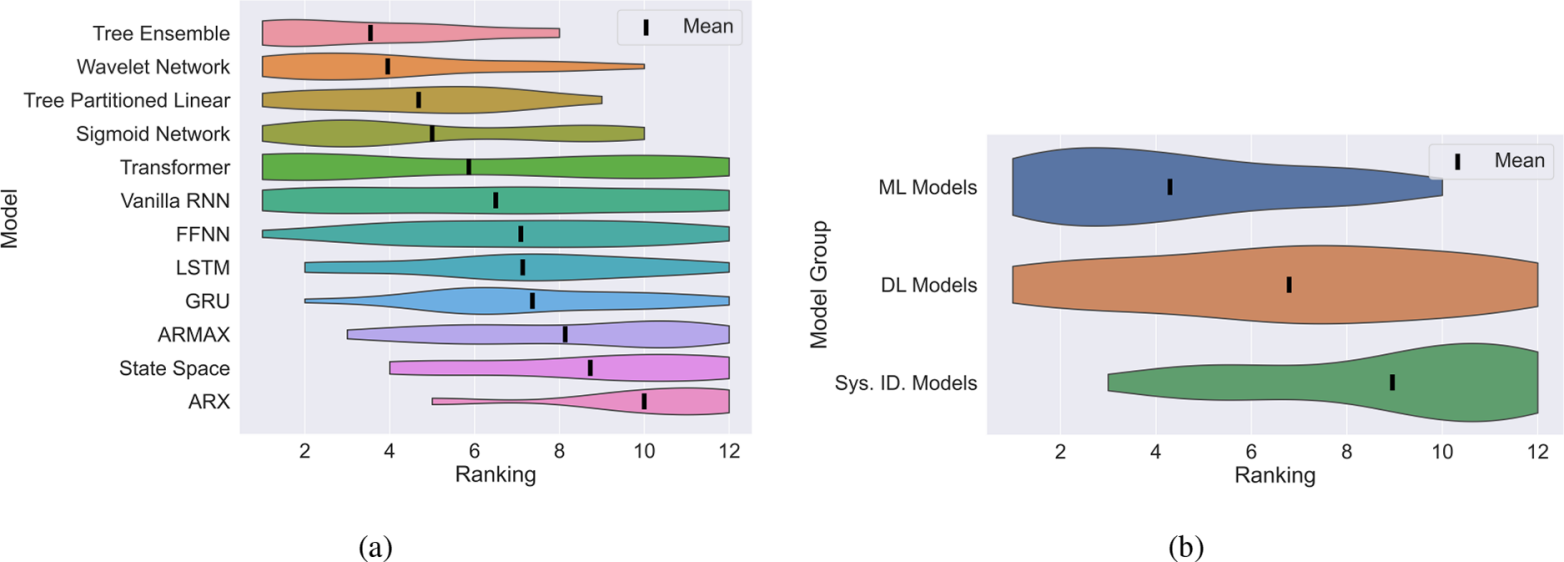
A ridge plot showing the ranking of the models based on their test MSE across the small and big data regime. (a): Individual models ranked. (b): Models grouped into system identification, ML or DL categories.

Our findings from both Sections 3.3.1 and 3.3.2 underscore the efficacy of ML models with balanced complexity (or what we refer to as “moderately” complex ML models) such as tree ensembles. They avoid the overparameterization and complexity of DL models (which makes them susceptible to long training times in general and overfitting in the small data regime in particular) while surpassing the predictive capabilities of simpler system identification models. We clearly demonstrate this by providing an overall ranking of the models based on their performance across both Sections 3.3.1 and 3.3.2 as depicted in Figure 16. Again, these results show that “ML Models” on average perform the best across all data regimes considered with the top three models belonging to this class. On the other hand, while “DL Models” perform well on average, but they have a large variance in their ranking as they perform extremely well in the big data regime (Figure 13) but then perform extremely poorly in the small data regime (Figure 9).

We find that our results corroborate recent trends reported within the ML community,^22,94^ where tree ensemble models have been shown to outshine DL models in tabular data settings like time-series prediction. However, our results go one step further and show that this trend is not unique to tree-ensemble models alone but to a number of ML models which strike the balance between model complexity and efficient training. With that being said, our results also suggest that in the big data regime, if efficient training is not a concern, then DL models such as transformers are undoubtedly the best performers in terms of prediction capabilities.

## 4 Conclusions

In this paper, we have undertaken a thorough and exhaustive comparison of system identification, ML, and DL models for the data-driven modeling of dynamical systems. Leveraging MLOps-inspired workflows we have systematically explored four key aspects as outlined in Section 2.1. Our findings shed light on pivotal factors influencing model selection and performance across varying data regimes. The main takeaway from our paper is that “trade-offs” are central throughout the entire modeling framework, from model selection to evaluation. At each stage, it is essential to first identify what these trade-offs are and then to choose algorithms or models that balance these trade-offs based on the specific requirements of the task at hand.

First, our investigation underscores the critical role of the search algorithm or hyperparameter optimization algorithm in model selection. Bayesian optimization, employing TPE, emerges as a balanced approach, effectively navigating the trade-off between selecting high-performing models and minimizing computational overhead.

Second, we emphasize the significance of model selection criteria in determining the quality of selected models. Our results indicate that k-fold cross-validation serves as a robust criterion, demonstrating consistent performance across diverse data regimes. Conversely, while information criteria falter in the small data regime, they are comparable to k-fold cross-validation in scenarios with abundant data, offering a computationally efficient alternative if required.

Finally, following a thorough model selection process, we identify ML models with balanced complexity, such as tree-partitioned linear models and tree ensembles, as the top performers across all data regimes. These models strike a delicate balance between predictive accuracy and training time, outperforming both overly simplistic system identification models and overly complex DL models. While DL models, like transformers, demonstrate exceptional predictive performance in the big data regime, they entail prolonged training times in general and are prone to significant overfitting in small data scenarios. On the other hand, simple linear-in-structure system identification models prove insufficient in capturing the full nonlinear dynamics of the systems under study. In contrast, moderately complex models such as single-layered wavelet networks and tree ensembles offer a compromise, effectively capturing system dynamics while maintaining computational efficiency. Overall, our findings underscore the nuanced interplay among model complexity, data regime considerations, and predictive performance, offering valuable insights for practitioners navigating the realm of dynamical system modeling.

## A AutoSID Framework Details

In this appendix, we describe the steps of the AutoSID framework outlined in Section 2.1 in detail. To accompany Figure 2, we also include Pseudocode 1 for reference.

### A.1 Input to the Framework

The framework is initialized by defining six different input sets:  and .  specifies the set of dynamical systems, defined by their system of equations, which are used to generate the time-series data for identification.  defines a particular dynamical system and its associated system of equations. For instance, this may be a CSTR. The full set of dynamical systems considered in this study are detailed in Section 2.2 and Appendix B.  signifies the range of sample sizes employed for training, where  denotes a specific number of samples, such as 1000 timesteps. Details on data generation, training, and testing allocation are discussed in Section 2.2.  defines the set of models to be evaluated, where  defines a particular model family, for instance, LSTM models. A particular instance of a model is only defined once the model structure is decided. The possible model structure or hyperparameters associated with each of the models in  is given by the set  where  is the set of possible hyperparameters for model . For example, for FFNN models, this set may include the possible number of layers, the number of nodes per layer, the activation function etc. The full set of models considered in this study, , as well as their associated hyperparameter/model structure choices are discussed in detail in Section 2.3.  comprises the collection of model selection search (hyperparameter optimization) algorithms utilized for exploring the hyperparameter space of a model.  represents a specific algorithm, such as the grid search method. Model selection and the search algorithms used will be further discussed in Section 2.4.  is the set of model selection criteria used to select the “best” model structure from the set of possible model structures.  is a particular criterion from this set with AIC being an example. Model selection criteria will also be discussed in Section 2.4.

### A.2 Output of the Framework

After performing model selection in step 4 and performance evaluation in step 5, the framework saves the full set of results to a database for postprocessing in order to analyze the results. Specifically, the following items are recorded after each iteration:

1. The system the data was generated from, .
2. The model which was fit to the data, .
3. The optimal hyperparameters/model structure for model  as assessed during model selection.
4. The number of training samples used, .
5. The search algorithm employed during model selection, .
6. The model selection criterion employed during model selection, .
7. The total model selection time.
8. The training MSE.
9. The testing MSE.
10. The total training time.

These results, for all combinations of the input sets, can then be used to analyze the results in order to investigate the four criteria mentioned in Section 2.1. More details on model performance evaluation can be found in Section 2.5.

### A.3 Step 1 and 7: Iterate through All Combinations

The framework iterates through steps 2 to 6 until every possible combination of the input sets , , , , and  is exhausted, generating results for each combination. For example, in one iteration,  might represent a CSTR system,  an ARX model,  being 500 training samples,  being the random search algorithm, and  being BIC. The next iteration may be identical, differing only in the model selection criterion i.e.  being 5-fold cross-validation. In this way, the results generated allow us to exhaustively compare the performance of the different models with respect to the four points mentioned in Section 2.1.

One should notice that all of the iterations are independent of each other and as such can be processed concurrently. This is a feature that we exploit in this study by running the iterations in parallel via SLURM job array parallelization using the Imperial College London HPC cluster.^27,28^ This parallelization was crucial for managing the considerable computational load posed by the 11,088 combinations under consideration. Executing steps 2 to 6 for each combination can be highly computationally intensive, particularly for DL models. SLURM job array parallelization optimizes efficiency by circumventing potential bottlenecks inherent in serial execution.^27^

### A.4 Step 3: Simulation of Dynamical Systems

After a particular system, , has been selected in step 2, the system is then simulated to generate the time-series data. A total of 40,000 timesteps are generated, but only a fraction of this is reserved for training while the remainder is used for testing. The number of samples used for training is given by  which is also selected in step 2.

Further details on all of the dynamical systems which are considered in this study as well as how the data is generated and how much of this is used for training and testing is discussed in Section 2.2.

### A.5 Step 4: Model Selection

Model selection is performed given a particular model, , its associated hyperparameters, , search algorithm,  and selection criteria,  from step 2 as well as the training data set generated from step 3. Model selection proceeds by finding the “optimal” model structure using the training data set whereby the search over the hyperparameters is enabled by the search algorithm while the selection criteria is used to assess what the “best” structure is. The optimal model structure and the total time taken for model selection is recorded. The optimal model structure is used in step 5 for performance evaluation (see Section 2.5), whereas the total model selection time is used for subsequent analysis to compare the different model selection search algorithms as will be shown in Section 3.1.

Further details on the models and their associated hyperparameter sets is considered in Section 2.3. Model selection, the different search algorithms and selection criteria are detailed in Section 2.4.

### A.6 Step 5: Performance Evaluation

After model selection in step 4, an optimal model structure, , for model, , is obtained. This model structure is imposed on the model which is then fit to the full training data set to give the training MSE, MSE_train_, and the total training time, *t*_training_. The trained model is then tested on the test data set to give the testing MSE, MSE_test_. These metrics are subsequently used to compare the performance of the different models to one another.

Further details on performance evaluation of the models and their subsequent comparison to each other is detailed in Section 2.5.

## B PSE Dynamical System Details

In this appendix, we describe in detail the 11 PSE dynamical systems used in this study to generate data for the system identification task. Specifically, the system of equations, parameter values, and the complexity associated with the dynamics as well as relevant sources of literature are presented.

### B.1 Second-Order System

In this section, we consider the general second-order dynamical system^95,96^ which consists of 2 state variables, 1 input variable, and 1 measured variable. The second-order system is described by the following set of differential equations:

3

where *x*_1_, *x*_2_ are the state variables, **x** = [*x*_1_, *x*_2_]^T^ ∈  and *u* is the input variable. We define the measured variable, which, in this case, is just a scalar, as *y* = *x*_1_. The remaining variables are treated as fixed parameters with their values given in Table 2. The system is simulated as per Section 2.2, with the input variable, *u*, defined by a series of random step inputs between a lower bound of 0 and 10.

**Table 2:** Table of Parameter Values Used for the Second-Order System

parameter	description	value	units
*K*	gain	1.0	–
τ	time constant	0.5	–
ζ	damping factor	0.25	–

We include the second-order system for a number of reasons. First, despite its simple form, it effectively describes various physical processes. For instance, in the PSE domain, it naturally emerges when mass capacities are arranged in series, such as two liquid storage tanks or a reactor feeding a storage tank, among other examples.^95^ Beyond PSE, the second-order system can also be used to describe the mechanical mass-damper system or the electrical RLC circuit.^96^ Moreover, despite its apparent simplicity, the second-order system exhibits rich and varied behavior, including oscillatory responses to step inputs and variations in damping depending on the parameter values. This complexity can pose challenges in identification.^5^

### B.2 Continuous Stirred Tank Reactor (CSTR)

In this section, we consider a CSTR system with cooling provided by water flowing through a coiled tube.^95,97^ The reactor houses an irreversible chemical reaction in which species A is converted to species B: *A* → *B*. The system consists of 2 state variables, 1 input variable and 2 measured variables. The system is described by the following set of differential equations:

4

where *C*_*A*_, the concentration of species *A* in the reactor, and *T*, the temperature of the reactor, are the state variables, **x** = [*C*_A_, *T*]^T^ ∈  while, *u* = *T*_*c*_, the cooling water temperature, is the input variable. In this case, we assume all states are observable and as such we define the measured variable to be **y** = **x**. The remaining variables are treated as fixed parameters with their values given in Table 3. The system is simulated as per Section 2.2, with the input variable, *u*, defined by a series of random step inputs between a lower bound of 275 and 325 K.

**Table 3:** Table of Parameter Values Used for the CSTR

parameter	description	value	units
*q*	feed flow rate	100	m^3^/s
*V*	reactor volume	100	m^3^
ρ	liquid density	1000	kg/m^3^
*C*_*p*_	specific heat capacity	239	J/kg K
Δ*H*_*R*_	specific heat of reaction	–5 × 10^4^	J/mol K
	activation energy over gas constant	8750	K
*k*	rate constant	7.2 × 10^10^	1/s
*UA*	heat transfer coefficient	4 × 10^4^	W/K
*T*_*f*_	feed temperature	350	K
*C*_*A*_*f*__	feed concentration of *A*	1.0	mol/m^3^

We include the CSTR system in our study as it represents a quintessential example of the dynamical systems explored in the PSE literature: highly nonlinear and complex, with potentially unstable dynamics depending on the exothermic or endothermic nature of the reaction. In the CSTR, the nonlinearities are particularly pronounced due to the presence of the state variable, *T*, in the exponential term. Furthermore, numerous theoretical, computational, and experimental investigations have shown that the CSTR system displays bifurcations,^98,99^ output multiplicity,^100^ chaotic behavior, and self-sustained oscillations^101,102^ depending on the region of the state space under consideration. Even in the simplest cases involving a single irreversible reaction, this rich behavior is evident.^98,95^ All of these phenomena contribute to the well-documented challenge of system identification for the CSTR system.^103^

### B.3 CSTR in Series with Recycle Loop

Building upon the complexity of the last section, we consider the same setup as in Section B.2 of a CSTR housing an irreversible chemical reaction, *A* → *B*, with cooling. However, now we consider two of these CSTR systems in series with a fraction of the output of the second CSTR being recycled back to the first reactor. The system is described in^104^ which is where we obtain the system of equations and the corresponding parameter values. The system consists of 4 state variables, 4 input variables, and 4 measured variables. The system is described by the following set of differential equations:

5

where *C*_*A*_1__ and *C*_*A*_2__, the concentrations of species A in reactor 1 and 2 respectively, and *T*_1_ and *T*_2_, the temperatures of reactor 1 and 2 respectively are the state variables, **x** = [*C*_A_1__, *C*_A_2__, *T*_1_, *T*_2_]^T^ ∈ . The cooling water temperatures of the two reactors as well as the feed and recycle flow rate are the input variables, **u** = [*T*_c1_, *T*_c2_, *q*_f_, *q*_r_]^T^ ∈ . As before, we assume all states are observable, **y** = **x**. The remaining variables are treated as fixed parameters with their values given in Table 4. These are obtained from.^104^ The system is simulated as per Section 2.2, with the input variable, **u**, defined by a series of random step inputs between a lower bound of [273 K, 273 K, 10 m^3^/s, 10 m^3^/s]^T^ and an upper bound of [325 K, 325 K, 40 m^3^/s, 50 m^3^/s]^T^.

**Table 4:** Table of Parameter Values Used for the CSTR with Recycle

parameter	description	value	units
*V*_1_	reactor volume 1	100	m^3^
*V*_2_	reactor volume 2	200	m^3^
ρ	liquid density	1050	kg/m^3^
*C*_*p*_	specific heat capacity	3766	J/kg K
Δ*H*_*R*_	specific heat of reaction	5.841 × 10^4^	J/mol K
	activation energy over gas constant	5550	K
*k*	rate constant	3.118 × 10^5^	1/s
*U*_1_*A*_1_	heat transfer coefficient of reactor 1	461	W/K
*U*_2_*A*_2_	heat transfer coefficient of reactor 2	732	W/K
*T*_*f*_	feed temperature	298	K
*C*_*A*_*f*__	feed concentration of *A*	1.0	mol/m^3^

The CSTRs in series, coupled with a recycle loop, introduce an additional layer of complexity to the challenges outlined in Section B.2. Specifically, the nonlinearities observed in a single CSTR are magnified by the interacting mass and thermal capacities when multiple reactors are arranged in series.^105,106^ Controlling such a system presents several significant practical challenges.^104,101^ Furthermore, the recycle loop introduces long-time dependencies on these interactions, resulting in substantial time delays, which are notoriously difficult for system identification.^107,108^

### B.4 Four Tank System

In this section, we consider the four-tank system as described in.^109,110,43^ The system consists of four tanks with two pumps used to control the liquid level in the tanks. Liquid from a reservoir is pumped to the tanks with a majority of the fluid being directed to the two upper tanks which drain into the two lower tanks while a smaller proportion of the flow is directed into the lower tanks directly. The system is composed of 4 state variables, 2 input variables, and 4 measured variables. The system is described by the following set of differential equations:

6

where the liquid level of the tanks are the state variables, **x** = [*h*_1_, *h*_2_, *h*_3_, *h*_4_]]^T^ ∈ . The pump voltages are the input variables, **u** = [*v*_1_, *v*_2_]^T^ ∈ . As before, we assume all states are observable, **y** = **x**. The remaining variables are treated as fixed parameters with their values given in Table 5. The system is simulated as per Section 2.2, with the input variable, **u**, defined by a series of random step inputs between a lower bound of [0V, 0V]^T^ and an upper bound of [10V, 10V]^T^.

**Table 5:** Table of Parameter Values Used for the Four Tank System

parameter	description	value	units
*g*	acceleration due to gravity	9.81	m/s^2^
γ_1_	fraction bypassed to Tank 1	0.2	–
γ_2_	fraction bypassed to Tank 2	0.2	–
*k*_1_	pump 1 gain	8.5 × 10^–4^	m^3^/V s
*k*_2_	pump 2 gain	9.5 × 10^–4^	m^3^/V s
*a*_1_	cross section area of tank 1 outlet	3.5 × 10^–3^	m^2^
*a*_2_	cross section area of tank 2 outlet	3.0 × 10^–3^	m^2^
*a*_3_	cross section area of tank 3 outlet	2.0 × 10^–3^	m^2^
*a*_4_	cross section area of tank 4 outlet	2.5 × 10^–3^	m^2^
*A*_1_	cross section area of tank 1	1.0	m^2^
*A*_2_	cross section area of tank 2	1.0	m^2^
*A*_3_	cross section area of tank 3	1.0	m^2^
*A*_4_	cross section area of tank 4	1.0	m^2^

The four-tank system is incorporated into this comparative study because it serves as a standard benchmark problem for identification and control tasks in the chemical engineering literature. Its complex, multivariate nonlinear dynamics, accentuated by the strongly interacting tank liquid capacities, make it an ideal testbed.^110,43^ Additionally, the four-tank system displays a unique dynamic property: it can be either a minimum phase or a nonminimum phase depending on the valve positions and pump flows to the tanks.^109^

### B.5 Continuous Binary Component Distillation Column

In this section, we consider the continuous distillation of a binary component system which is well described and surveyed in the literature.^97,111−113^ The system consists of a distillation column operated at constant liquid holdup, fed with a saturated liquid composed of two components, of differing volatility, which we refer to as A and B respectively. The distillation column is composed of 7 plates, numbered from top to bottom, including the feed plate at position 4 as well as a reflux drum and a reboiler. Consequently, the system is described by 9 state variables, 2 input variables, and 2 measured variables. The system of differential equations consists of a material balance for the more volatile component, A, at five main regions within the column. Starting from the top and moving down; A balance at the reflux drum:

7

where *x*_*D*_ is the liquid mole fraction of component A in the reflux drum i.e. the distillate mole fraction. *y*_1_ is the vapor mole fraction of component A from plate 1. *M*_*D*_ is the number of moles of liquid in the reflux drum. *V* is the vapor molar flow rate in the rectifying section. *L* is the liquid molar flow rate in the rectifying section. Finally, *D* is the distillate molar flow rate.

A balance for plate *n*, in the rectifying section, above the feed stream

8

where *L* and *V* are defined as before. *M* is the number of moles of liquid retained on the current plate. *x*_*n*–1_ and *x*_*n*_ are the liquid mole fractions of component A from the plate above, *n* – 1, and on the current plate, *n*. Similarly *y*_*n*+1_ and *y*_*n*_ are the vapor mole fractions of component A from the plate below, *n* + 1, and on the current current plate.

A balance for the feed plate, *f*:

9

where *M*, *L* and *V* are defined as before. *L′* is the liquid molar flow rate from the stripping section. *V ′* is the vapor molar flow rate from the stripping section. *F* is the feed molar flow rate. *x*_feed_ is the liquid mole fraction of A in the feed stream. *x*_*f*_ and *x*_*f*–1_ are the liquid mole fractions of A on the feed plate, *f*, and the plate above it, *f* – 1, respectively. Similarly, *y*_*f*_ and *y*_*f*+1_ are the vapor mole fractions of component A on the feed plate, and the plate below it, *f* + 1, respectively.

A balance for plate *m*, in the stripping section, below the feed stream:

10

where all variables are defined as they were previously.

Finally, a balance for the reboiler:

11

where *L*′ and *V*′ are defined as before. *M*_*B*_ is the number of moles of liquid in the reboiler. *x*_*B*_ is the liquid mole fraction of *A* in the reboiler i.e., the bottom composition. *y*_*B*_ is the vapor mole fraction of *A* rising from the reboiler. Finally, *x*_7_ is the liquid mole fraction of *A* dropping from the final plate in the distillation column, plate 7.

Additionally, the system requires several algebraic equations describing the total material balance over the column. The liquid and vapor molar flow rates in the rectifying section:

12

where *D*, *L,* and *V* are defined as before. *R* is the reflux ratio.

The liquid and vapor molar flow rates in the stripping section:

13

where *L*′, *V*′, *L*, *V,* and *F* are defined as before. *q* is the feed “quality” where in this case a saturated liquid feed is considered, such that *q* = 1.

Finally, the system also requires the equilibrium relationship between the liquid and vapor at any stage, *i*:

14

where *x*_*i*_ and *y*_*i*_ are the liquid and vapor mole fractions at stage *i* respectively. α is the relative volatility of the most volatile component, A, to the less volatile component, B.

The state variables are component A’s liquid mole fractions at each stage, **x** ∈ (9) while the feed flow rate and reflux ratio are the input variables, **u** = [*F*, *R*]^T^ ∈ . In this case, we assume the only measured variables are the distillate and bottom compositions, **y** = [*x*_D_, *x*_B_]^T^ ∈ . All other variables are treated as fixed parameters with their values given in Table 6. The system is simulated as per Section 2.2, with the input variable, **u**, defined by a series of random step inputs between a lower bound of [10.0 mol/h, 2.0]^T^ and an upper bound of [100.0 mol/h, 10.0]^T^.

**Table 6:** Table of Parameter Values Used for the Distillation Column

parameter	description	value	units
*M*	moles of liquid retained in column	2000	mol
*D*	distillate molar flow rate	100	mol/h
*q*	feed quality	1.0	–
α	relative volatility of component A to B	5.0	–
*x*_feed_	feed composition of component A	0.2	–

Just as the CSTR system epitomizes the intricate dynamics encountered by chemical engineers, the distillation column stands as a quintessential example of distributed parameter systems found commonly in chemical engineering.^97,112,114^ These systems, theoretically infinite-dimensional, are typically described by partial differential equations (PDEs) detailing their spatial and temporal evolution.^115,116^ Consequently, spatial discretization methods such as finite differences or finite elements are employed. This often leads to the development of nonlinear high-order models, sometimes necessitating model-order reduction methods for severe cases.^117^ Despite being standard unit operations in PSE, accurately modeling or performing system identification of distillation columns for successful model-based control remains challenging.^112^ Even for the simplest cases involving binary components and ideal thermodynamics, columns may exhibit multiple steady states or catastrophic instabilities.^118,119^ Challenges intensify with nonideal mixtures, azeotropes, or unconventional configurations like linked columns or divided walls.^111,120−124^ Consequently, practitioners often rely on basic first-principles models, which are normally poor approximations of the underlying system.^112,125^

### B.6 Multistage Countercurrent Extraction Cascade

In this section, we consider the multistage countercurrent liquid–liquid extraction cascade system as detailed in a previous study.^97^ The system consists of two immiscible liquids, which we denote as *L* and *G* respectively. A solute is preferentially transferred from phase *L* to phase *G*. The mass transfer occurs via an extraction cascade whereby 5 extraction stages are placed in series with the two liquid phases flowing countercurrent to each other in order to maximize the extraction efficiency. The system has 10 state variables, 2 input variables, and 2 measured variables. The system is described by the following system of differential equations which expresses a solute balance in each phase for every stage, *i*:

15

where the state variables are the concentrations of the solute in phase *L* and phase *G* at each stage, *X*_*i*_ and *Y*_*i*_, **x** ∈ .^10^ The input variables are defined to be the flow rate of the two phases through the extraction cascade, **y** = [*L*, *G*]^T^ ∈ . We assume that the only measured variables are the outlet concentrations of the solute in each of the phases, **y** = [*X*_5_, *Y*_1_]^T^ ∈ . The remaining variables are treated as fixed parameters with their values given in Table 7. The system is simulated as per Section 2.2, with the input variable, **u**, defined by a series of random step inputs between a lower bound of [1.0 m^3^/h, 1.0 m^3^/h]^T^ and an upper bound of [5.0 m^3^/h, 5.0 m^3^/h]^T^.

**Table 7:** Table of Parameter Values Used for the Extraction Cascade

parameter	description	value	units
*V*_*L*_	liquid volume of each stage	5.0	m^3^
*V*_*G*_	gas volume of each stage	5.0	m^3^
*K*_*La*_	mass transfer constant	5.0	1/h
*X*_0_	feed concentration of solute in liquid	0.6	–
*Y*_6_	feed concentration of solute in gas	0.05	–
*m*	equilibrium constant	1.2	–

The extraction cascade presents numerous complexities that make identification challenging. Similar to distillation columns, extraction cascades are distributed parameter systems with spatially evolving dynamics. The system dynamics involve various competing and opposing effects, including multiphase equilibrium, mass transfer transience, and hydrodynamic phenomena like backmixing.^97,126,127^ These opposing dynamic behaviors can complicate identification, particularly with limited data that may not fully capture the nonlinear characteristics or if the system has not been appropriately excited by the input signals.^128−130^ Additionally, depending on the specific extraction mechanism or cascade configuration, extraction cascades can exhibit a wide range of dynamic behaviors, from slow dynamics dominated by near-equilibrium conditions in high-capacity, long-residence-time cascades to rapidly evolving dynamics in low-volume, high-throughput devices.^97,129^

### B.7 Multistage Countercurrent Extraction Cascade with a Slow Chemical Reaction

Building on Section B.6, in this section we consider the same setup as before but include an additional layer of complexity in the form of a slow chemical reaction occurring within the cascade. As before, the system consists of two immiscible liquids, which we denote as *L* and *G* respectively. In this case, we consider a specific solute which we call A. As before, A is transferred preferentially from phase *L* to phase *G*. However, in this case, we assume that solute A reacts with a second component B (in phase *G*) to form an inert product C: *A* + *B* → *C*. It should be noted that components B and C are immisible in phase *L* and therefore remain in phase *G*. For this reason, phase *L* only contains component A while phase *G* contains all three components. The mass transfer occurs via 5 extraction stages placed in series with the two liquid phases flowing countercurrent to each other. The system has 20 state variables, 2 input variables, and 2 measured variables. As before, the system is described by the following system of differential equations which expresses a component balance in each phase for every stage, *i*:

16

where the state variables are the concentrations of solute A in phase *L* and phase *G* at each stage, *X*_*A*_*i*__ and *Y*_*A*_*i*__, as well as the concentrations of component B in phase *G* at each stage, *Y*_*B*_*i*__, and the concentrations of component C in phase *G* at each stage, *Y*_*C*_*i*__ i.e. **x** ∈ .^20^ The input variables are defined to be the flow rate of the two phases through the extraction cascade, **u** = [*L*, *G*]^T^ ∈ . As before, we assume that the only measured variables are the outlet concentrations of solute A in each of the phases, **y** = [*X*_A_5__, *Y*_A_1__]^T^ ∈ . The remaining variables are treated as fixed parameters with their values given in Table 8. The system is simulated as per Section 2.2, with the input variable, **u**, defined by a series of random step inputs between a lower bound of [1.0 m^3^/h, 1.0 m^3^/h]^T^ and an upper bound of [5.0 m^3^/h, 5.0 m^3^/h]^T^.

**Table 8:** Table of Parameter Values Used for the Extraction Cascade With Reaction

parameter	description	value	units
*V*_*L*_	liquid volume of each stage	5.0	m^3^
*V*_*G*_	gas volume of each stage	5.0	m^3^
*K*_*La*_	mass transfer constant	0.01	1/h
*k*	rate constant	0.1	1/h
*X*_*A*_0__	feed concentration of A in liquid	0.85	–
*Y*_*A*_6__	feed concentration of A in gas	0.05	–
*Y*_*B*_6__	feed concentration of B in gas	0.9	–
*Y*_*C*_6__	feed concentration of C in gas	0.05	–
*m*	equilibrium constant	1.2	–

This system suffers the same complexities that make identification challenging for the standard extraction cascade described in Section B.6. Additionally, one must also contend with the complexities introduced by chemical reaction dynamics as was introduced in Section B.2. The original investigators of the above model,^97^ illustrate these challenges, revealing sustained oscillatory behavior due to the inclusion of reaction dynamics. Furthermore, the inclusion of a chemical reaction, even if slow-evolving, introduces a distinct set of dynamic behaviors characterized by an additional opposing effect in the cascade. Specifically, as the transferred solute, A, is slowly consumed in phase *G*, the affinity for mass transfer from phase *L* to *G* varies depending on the rate of the reaction which in turn depends on the concentration of the components in phase *G* itself. This interplay can induce sustained oscillations and substantial time delays, as noted by^97^ and.^128^

### B.8 Nitrification in a Fluidized Biofilm Sand Bed Reactor

In this section we consider the nitrification process taking place in a fluidized biofilm sand bed reactor. The process is typically employed for biological wastewater treatment in order to remove Nitrogen, in the form of Ammonium, from water.^131^ In the reactor, Ammonium undergoes sequential oxidation (under the action of biomass contained in the biofilm of the reactor) to nitrite and then nitrate according to the following reactions:

17

The outlet, rich in Nitrite and Nitrate as well as any unreacted Ammonium, is passed to an absorber which attempts to remove the products from the stream. At the same time, the stream is enriched with oxygen by mass transfer from the air. Oxygen is needed for the nitrification reaction as shown in eq 17. In this stream, with unabsorbed Nitrite and Nitrate ions, the unreacted ammonium as well as newly dissolved oxygen is then recycled back to the fluidized biofilm reactor in order to convert as much Ammonium as possible.

The simulation model employed is loosely based on the early work of,^132^ however, is supplemented by more recent contributions in the form of.^97,133,134^ The fluidized biofilm reactor is modeled by discretizing the reactor into three control volumes and treating each control volume as a lumped parameter system (effectively as three tanks in series). The system has 16 state variables, 5 input variables, and 3 measured variables. The system is described by the following set of differential equations detailing the material balance of the four components (ammonium, nitrite, nitrate, and oxygen) in the three reactor stages as well as the absorber. Note that for notational convenience, the nitrogen compounds are indexed as substrate 1 (ammonium), 2 (nitrite), and 3 (nitrate).

For reactor stage *n*, we have

18

where *S*_*i*_*n*__ and *S*_*i*_*n*–1__ are the concentrations of substrate *i* (where *i* = 1, 2, 3, corresponds to ammonium, nitrite, and nitrate) in stage *n* and *n* – 1 respectively. *O*_*n*_ and *O*_*n*–1_ correspond to the concentrations of oxyen in stage *n* and *n* – 1 respectively. *q*_*r*_ is the recycle flow rate. *V* is the volume of each reactor stage. The upcoming variables are all related to the double Michaelis–Menten rate expressions in eq 18, for more details refer to.^97,132−134^*v*_max*j*_ is the maximum velocity for reaction *j* in eq 18. *K*_*j*_ is the saturation constant for Ammonia for reaction *j* in eq 18.

For the absorber, we have

19

where *q*_*r*_ and *V* are defined as before. *S*_*i*_3__, *S*_*i*_*A*__, and *S*_*i*_*F*__ are the concentrations of substrate *i* in the final reaction stage, stage 3, the absorber and the feed to the absorber, respectively. *q* is the feed flow rate to the absorber while *V*_*A*_ is the volume of the absorber unit. *O*_3_, *O*_*A,*_ and *O*_Air_ are the concentrations of Oxygen in the final reaction stage, the absorber, and in the air, respectively. Finally, *K*_*La*_ is the mass transfer coefficient while *m* is the equilibrium constant which dictates the equilibrium relationship between the absorbed oxygen and the oxygen in the air.

The state variables are the substrate and oxygen concentrations in each of the reaction stages and in the absorber, **x** ∈ (16) while the recycle flow rate, the feed flow rate to the absorber as well as the feed substrate concentrations to the absorber are the input variables, **u** = [*q*_r_, *q*, *S*_1_F__, *S*_2_F__, *S*_3_F__]^T^ ∈ . The measured variables are assumed to be the concentrations of the substrates in the outlet of the reactor, **y** = [*S*_1_3__, *S*_2_3__]^T^ ∈ . All other variables are treated as fixed parameters with their values given in Table 9. The system is simulated as per Section 2.2, with the input variable, **u**, defined by a series of random step inputs between a lower bound of [0.0 L/h, 1.0 L/h, 0.5 mg/L, 0.5 mg/L, 0.05 mg/L]^T^ and an upper bound of [10.0 L/h, 30.0 L/h, 1.0 mg/L, 1.0 mg/L, 1.0 mg/L]^T^.

**Table 9:** Table of Parameter Values Used for the Fluidized Biofilm Reactor

parameter	description	value	units
*V*	volume of reactor stage	10.0	L
*V*_*A*_	volume of absorber	15.0	L
*K*_*La*_	mass transfer constant	1.5	1/h
*k*	rate constant	0.1	1/h
*O*_Air_	concentration of oxygen in air	300	mg/L
*K*_1_	ammonia saturation constant for reaction 1	0.5	mg/L
*K*_2_	ammonia saturation constant for reaction 2	0.1	mg/L
*K*_*O*1_	oxygen saturation constant for reaction 1	1.5	mg/L
*K*_*O*2_	oxygen saturation constant for reaction 2	0.5	mg/L
*v*_max1_	maximum velocity through bed for reaction 1	0.8	mg/L h
*v*_max2_	maximum velocity through bed for reaction 2	1.0	mg/L h
*m*	equilibrium constant	0.5	–

The inclusion of the biofilm reactor in this study serves multiple purposes. First, the nitrification of Ammonium is a critical process in wastewater treatment, necessitating the development of model-based control strategies for its efficient operation, as recognized by various practitioners.^133,134,131^ Unfortunately, due to the complexity of bioprocesses, such strategies are seldom employed in practice, leading to reliance on heuristic and ad-hoc operating strategies.^134,131^ Accurate model development presents a significant challenge, as most first-principles models lack the required precision.^133^ Conversely, data-driven modeling approaches based on system identification and ML methods show promise.^135−138^ Hence, our study aims to explore various data-driven approaches for bioprocesses. However, bioprocesses pose numerous complexities for data-driven modeling. Similar to the discussion in Section B.6, they exhibit competing dynamic behaviors evolving at different lengths and time scales, often complicated by large recycle loops necessary for sufficient reactant conversion, resulting in long time dependencies and delays.^97,136,137,107^ These complexities include reaction dynamics, transient mass transfer, and hydrodynamic effects influenced by the unique rheology of bioprocesses, among other factors.^139,140^ Such intricacies lead to the typical challenges associated with identifying nonlinear systems; for instance, reacting biosystems may exhibit both stable and unstable limit cycles,^141^ along with sustained oscillations.^142^ Consequently, identifying and controlling these systems is a formidable task.

### B.9 Shell and Tube Heat Exchanger

In this section, we consider a fundamental unit operation in chemical engineering via the shell and tube heat exchanger with the model employed based on several sources.^97,143−145^ In the shell and tube heat exchanger, the hot fluid is assumed to be on the tube side which flows counter-current to the cold fluid on the shell side. The finite difference method is used to spatially discretize the heat exchanger into 8 sections. Consequently, the system consists of 24 state variables, 4 input variables, and 2 measured variables described by the following set of differential equations for each section, *i*:

20

where *T*_*t*_*i*__, *T*_*s*_*i*__, and *T*_*m*_*i*__ are the temperatures of the tube-side liquid, the shell-side liquid, and the metal wall separating the liquids for stage *i* respectively. These temperatures for all of the stages define the state of the system, **x** ∈ .^24^ The inputs to the system are the tube-side flow rate, *q*_*t*_, the shell-side flow rate, *q*_*s*_, the tube-side inlet temperature, *T*_*t*_0__, and the shell-side inlet temperature, *T*_s_9__: **u** = [*q*_t_, *q*_s_, *T*_t_0__, *T*_s_9__, ]^T^ ∈ . Finally, we assume that only the outlet temperature of the two sides can be measured, **y** = [*T*_t_9__, *T*_s_0__]^T^ ∈ . All other variables are treated as fixed parameters with their values given in Table 10. The system is simulated as per Section 2.2, with the input variable, **u**, defined by a series of random step inputs between a lower bound of [10.0 m^3^/h, 20.0 m^3^/h, 353 K, 293 K]^T^ and an upper bound of [50.0 m^3^/h, 200.0 m^3^/h, 423 K, 323 K]^T^.

**Table 10:** Table of Parameter Values Used for the Heat Exchanger

parameter	description	value	units
*U*_*tm*_	metal tube overall heat transfer coefficient	1.5	kW/m^2^ K
*U*_*ms*_	metal sheet overall heat transfer coefficient	0.5	kW/m^2^ K
*L*	segment length	2	m
*D*_*t*_	tube wall internal diameter	0.05	m
*D*_*m*_	metal wall outside diameter	0.052	m
*D*_*s*_	shell wall diameter	0.75	m
*C*_*p*_*t*__	tube-side fluid specific heat capacity	4.18	kJ/kg K
*C*_*p*_*m*__	metal wall specific heat capacity	0.5	kJ/kg K
*C*_*p*_*s*__	shell-side fluid specific heat capacity	1.70	kJ/kg K
ρ_*t*_	tube-side fluid density	1000	kg/m^3^
ρ_*m*_	metal density	7850	kg/m^3^
ρ_*s*_	shell-side fluid density	800	kg/m^3^

The inclusion of the shell and tube heat exchanger in this study is motivated by its significance as one of the most crucial unit operations in chemical engineering as a heating process. To develop plantwide model-based control strategies for most process systems, an accurate dynamic model of heat exchangers is essential.^97,145,146^ Additionally, the transient behavior of heat exchangers is of great interest, particularly during start-up and shut-down, where load changes can lead to instabilities such as pressure build-ups, rapid phase changes, and overall poor system control.^144−148^ A detailed analysis of modeling and control challenges is provided in a previous study,^145^ where issues such as time delays and long exchanger residence times are highlighted, complicating both system identification and control tasks.

### B.10 Homogeneous Free-Radical Polymerization

In this section, we consider the homogeneous free-radical polymerization reaction in a CSTR based on the models reported earlier,^97,149^ which is a simplification of the full large-scale model previously published.^150^ Free-radical polymerization is an industrially important process as it is commonly employed in the mass manufacturing of a variety of polymers and polymer-based products.^151,150^ The polymerization of free radicals proceeds as a sequence of three distinct steps termed initiation, addition, and termination within a CSTR. At a high level, the initiation step involves the formation of free radicals from an initiator component. The free radicals are then subjected to addition or propagation whereby they are used to create monomers which add to the growing polymer chain. Finally, in termination, the propagation stage is inhibited when the degree of polymerization is obtained. For the interested reader, a more detailed and thorough discussion of the reaction mechanism and kinetics is described in the aforementioned references. For the model we consider in this paper, the system consists of 3 state variables, 4 input variables, and 1 measured variable. The system is described by the following set of algebraic and differential equations which gives an overall energy balance, a component balance for the Monomer and Initiator, and the average chain length of the polymer following termination.

21

The kinetic rate coefficients for the decomposition of the initiator, *k*_*d*_, the propagation reaction, *k*_*p*_, as well as the termination reaction, *k*_*t*_, are all defined by an Arrhenius equation temperature dependency:

22

Finally, the average chain length of the polymer depends on the extent of the different reactions and is given by

23

where the temperature of the reactor, the monomer concentration, and the initiator concentrations are treated as the state variables, **x** = [*T*, *M*, *I*]^T^ ∈  while the flow rate, temperature, monomer concentration, and initiator concentration of the feed stream are defined as the input variables, **u** = [*F*, *T*_f_, *M*_f_, *I*_f_]^T^ ∈ . It is assumed that the only measured variable is the average chain length of the polymer, *y* = *L*_*x*_. All other variables are treated as fixed parameters with their values given in Table 11. The system is simulated as per Section 2.2, with the input variable, **u**, defined by a series of random step inputs between a lower bound of [5.0 m^3^/h, 300 K, 5000.0 mol/m^3^, 10.0 mol/m^3^]^T^ and an upper bound of [20.0 m^3^/h, 400 K, 5000.0 mol/m^3^, 200.0 mol/m^3^]^T^.

**Table 11:** Table of Parameter Values Used for the Polymerization Reaction

parameter	description	value	units
*A*_*d*_	initiation pre-exponential factor	4.0 × 10^10^	1/s
*A*_*p*_	propagation pre-exponential factor	6.0 × 10^10^	1/s
*A*_*t*_	termination pre-exponential factor	9.0 × 10^10^	1/s
	initiation activation energy over gas constant	8500	K
	propagation activation energy over gas constant	7750	K
	termination activation energy over gas constant	8250	K
Δ*H*_*R*_	specific heat of reaction	–3 × 10^4^	J/mol
*C*_*p*_	fluid mixture heat capacity	2.0	kJ/kg K
ρ	fluid mixture density	1200	kg/m^3^
*V*	reactor volume	50	m^3^
*f*	reactivity fraction of free radicals	0.5	–

We include the free-radical polymerization reaction in this study for several reasons. First, as extensively discussed in,^150^ the three distinct reaction stages of initiation, addition, and termination, exhibit complex nonlinear dependencies, presenting challenges akin to those mentioned in Section B.2 for reacting systems. However, specific to polymerization reactions, the competing dynamics of these stages give rise to the “gel” effect: At high degrees of polymerization, the reaction dynamics lead to multiple steady states and oscillations, causing a marked drop-off in the degree of polymerization. This phenomenon is well-documented computationally and experimentally.^97,150,152^ For instance,^150,153^ develop a model demonstrating hysteresis behavior due to multiple steady states resulting from the “gel” effect, consistent with experimental observations.^154^ In fact, due to the extremely rich nonlinear dynamics exhibited by such reactions, as well as their industrial importance, a multipart series of papers was released over the course of several years with the intention of improving the modeling, control, and operation of polymerization reactions. Moreover, polymerization reaction dynamics are frequently examined in large-scale system benchmark studies in the chemical engineering literature owing to its large number of possible system states.^155^ This is because, when considering the possible polymer chain lengths as a state of the system, there can be over a thousand system states depending on the exact polymer being considered. In this study, we employ simplified models from^97,149^ to simulate the system, utilizing average chain length for simplicity, as this model still gives rise to the many complexities discussed earlier.

### B.11 Plantwide Process System

For the final system, we include in this study, we consider a large-scale plantwide process system as described by the model in a previous study.^156^ The system consists of a reactor, storage tank, and flash tank as well as a recycle loop from the flash tank back to the reactor. The system consists of three components which we denote as A, B, and C. A is the feedstock to the system while B is the desired product and C is an undesired product. The reactor houses a two-stage irreversible liquid reaction where A → B → C. The reactor effluent is pumped to a storage tank before being sent to the flash separator. It is assumed that component A is more volatile than component B while component C is nonvolatile. Consequently, the flash separator is used to flash the volatile components from the separator feed stream such that the vapor top stream, composed of components A and B, is cooled and recycled back to the reactor while the bottom liquid stream, composed of unreacted A, product B, and undesired product C is pumped away to be processed further. The system consists of 12 state variables, 5 input variables, and 3 measured variables. The system is described by the following set of algebraic and differential equations.

A total material balance for the reactor:

24

where *H*_*R*_ is the reactor level, ρ is the reactor liquid density (which is assumed to be the same liquid density across the whole system), *A*_*R*_ is the cross-sectional area of the reactor, *F*_*f*_ is the feed flow rate to the reactor, *D* is the distillate flow rate from the flash separator recycled back to the reactor, and finally, *F*_*R*_ is the reactor effluent flow rate.

A component balance for the reactor:

25

where *H*_*R*_, ρ, *A*_*R*_, *F*_*f*_, *D*, and *F*_*R*_ are defined as they were previously. *x*_*Af*_ is the liquid mole fraction of component A in the feed stream. *x*_*AR*_, *x*_*BR*_, and *x*_*CR*_ are the liquid mole fraction of components A, B, and C in the reactor. *x*_*AD*_ and *x*_*BD*_ are the liquid mole fractions of components A and B in the distillate stream from the flash separator. Finally, *k*_*A*_ and *k*_*B*_ are the rate constants for the two chemical reactions.

A total material balance for the storage tank:

26

where ρ is defined as previously. *H*_*M*_ is the storage tank level, *A*_*M*_ is the cross-sectional area of the storage tank. *F*_*R*_ is the reactor outlet flow rate. *F*_*M*_ is the outlet flow rate of the storage tank as well as the inlet flow rate to the separator.

A component balance for the storage tank:

27

where ρ, *F*_*R*_, *A*_*M*_, *H*_*M*_, *x*_*AR*_, *x*_*BR*_, and *x*_*CR*_ are defined as they were previously. *x*_*AM*_, *x*_*BM*_, and *x*_*CM*_ are the liquid mole fractions of components A, B, and C in the storage tank.

A total material balance for the separator:

28

where ρ, *F*_*M*_, and *D* are defined as they were previously. *H*_*S*_ is the is the separator level, *A*_*S*_ is the cross-sectional area of the separator and *B* is the bottom flow rate from the separator.

A component balance for the separator:

29

where ρ, *F*_*M*_, *A*_*S*_, *H*_*S*_, *D*, *x*_*AM*_, *x*_*BM*_, and *x*_*CM*_ are defined as they were previously while *x*_*AS*_, *x*_*BS*_, and *x*_*CS*_ are the liquid mole fractions exiting the bottom of the flash separator.

It should be noted that the recycle stream, which is composed of the distillate liquid compositions following cooling, can be found from the bottom compositions using the relative volatility of component A with respect to component B (denoted α):

30

The state variables are the liquid mole fractions of component A, B and C throughout the process as well as the level of the liquid in the reactor, storage tank and separator, **x** ∈ .^12^ The input variables are the feed flow rate to the reactor, the outlet flow of the reactor, the outlet flow rate of the storage tank (or similarly the inlet flow to the separator) as well as the bottoms and distillate flow rate of the separator, **u** = [*F*_f_, *F*_R_, *F*_M_, *B*, *D*]^T^ ∈ . As the original paper, we assume the only measured variables are the bottoms liquid compositions and the recycled distillate liquid compositions respectively, **y** = [*x*_cAS_, *x*_BS_, *x*_AD_, *x*_BD_]^T^ ∈ . All other variables are treated as fixed parameters with their values given in Table 12. The system is simulated as per Section 2.2, with the input variable, **u**, defined by a series of random step inputs between a lower bound of [50.0 kmol/h, 50.0 kmol/h, 50.0 kmol/h, 50.0 kmol/h, 00.0 kmol/h,]^T^ and an upper bound of [500.0 kmol/h, 500.0 kmol/h, 500.0 kmol/h, 500.0 kmol/h, 500.0 kmol/h,]^T^.

**Table 12:** Table of Parameter Values Used for the Plantwide System

parameter	description	value	units
*A*_*R*_	reactor cross sectional area	5	m^2^
*A*_*M*_	storage tank cross sectional area	10	m^2^
*A*_*S*_	separator cross sectional area	5	m^2^
*k*_*A*_	reaction 1 rate constant	0.0167	1/h
*k*_*B*_	reaction 2 rate constant	0.0167	1/h
ρ	molar density	50	kmol/m^3^
α	relative volatility of component A to B	90	–

**Table 13:** Table of Possible Hyperparameters and Their Ranges for ARX Models

hyperparameter	variable type	range/values
input model order	discrete	1–15
output model order	discrete	1–15

**Table 14:** Table of Possible Hyperparameters and Their Ranges for ARMAX Models

hyperparameter	variable type	range/values
input model order	discrete	1–15
output model order	discrete	1–15
white noise MA order	discrete	1–15

**Table 15:** Table of Possible Hyperparameters and Their Ranges for State-Space Models

hyperparameter	variable type	range/values
model order	discrete	1–15

**Table 16:** Table of Possible Hyperparameters and Their Ranges for Tree-Partitioned Linear Models

hyperparameter	variable type	range/values
input model order	discrete	1–15
output model order	discrete	1–15
tree nodes	discrete	1–15

**Table 17:** Table of Possible Hyperparameters and Their Ranges for Single-Layered Sigmoid Network Models

hyperparameter	variable type	range/values
input model order	discrete	1–15
output model order	discrete	1–15
sigmoid nodes	discrete	1–15

**Table 18:** Table of Possible Hyperparameters and Their Ranges for Single-Layered Wavelet Network Models

hyperparameter	variable type	range/values
input model order	discrete	1–15
output model order	discrete	1–15
wavelet nodes	discrete	1–15

**Table 19:** Table of Possible Hyperparameters and Their Ranges for Regression Tree Ensemble Models

hyperparameter	variable type	range/values
input model order	discrete	1–15
output model order	discrete	1–15
fitting method	categorical	“Bagging”, “Boosting”
number of trees/boosting stages	discrete	100–1000
maximum tree depth	discrete	5–30

**Table 20:** Table of Possible Hyperparameters and Their Ranges for FFNN Models

hyperparameter	variable type	range/values
input model order	discrete	1–15
output model order	discrete	1–15
network layers	discrete	1–5
neurons per layer	discrete	2–10
activation function	categorical	“ReLU”, “Leaky ReLU”, “Sigmoid”, “Tanh”
initial learning rate	continuous	0.001–0.1

**Table 21:** Table of Possible Hyperparameters and Their Ranges for Vanilla RNN Models

hyperparameter	variable type	range/values
input model order	discrete	1–15
output model order	discrete	1–15
network layers	discrete	1–5
units per layer	discrete	16–512
activation function	categorical	“ReLU”, “Tanh”
initial learning rate	continuous	0.001–0.1

**Table 22:** Table of Possible Hyperparameters and Their Ranges for LSTM and GRU Models

hyperparameter	variable type	range/values
input model order	discrete	1–15
output model order	discrete	1–15
network layers	discrete	1–5
units per layer	discrete	16–512
activation function	categorical	“ReLU”, “Tanh”
recurrent dropout	continuous	0.2–0.5
initial learning rate	continuous	0.001–0.1

**Table 23:** Table of Possible Hyperparameters and Their Ranges for Transformer Models

hyperparameter	variable type	range/values
maximum input model order	discrete	1–15
maximum output model order	discrete	1–15
encoder/decoder layers	discrete	6–24
embedding size	discrete	64–1024
attention head size	discrete	2–32
feedforward network size	discrete	128–2048
dropout	continuous	0.1–0.3

We include the plantwide process system because it encapsulates the complexities discussed in previous sections: reaction dynamics (Sections B.2, B.3, B.7, B.8); challenges associated with recycle loops and their introduced time delays and dependencies (Sections B.3, B.4, B.8); interacting tank capacities in series (Sections B.1, B.4); and separation processes (Section B.5). This system could be further complicated by additional unit operations such as heating or more complex separation or purification units. Fundamentally, it represents the type of large-scale process systems that chemical engineers must model, control, optimize, and ultimately operate. However, as discussed in the original paper and other sources,^156,113,157^ these tasks are incredibly challenging on a plantwide scale for two primary reasons. First, the individual components comprising the process have their own intricate dynamics (as we have seen throughout Section 2.2) that must now be considered at a large scale. Indeed, an unwanted byproduct of this, is that the combination and interaction of these components can lead to unstable (and potentially chaotic) phenomena, such as the “Snowball” effect, where minor changes in the feed result in significant changes in process conditions.^156,113,157^ As one can imagine, these challenges make system identification on a plantwide scale an inevitably demanding task.

## C Data-Driven Model Hyperparameters

This appendix contains the table of possible hyperparameter choices and their range of values for each of the data-driven models outlined in Section 2.3.
